# Decoding spatiotemporal dynamics of suspended sediment and vegetation in shallow reservoirs with Sentinel-2 and ANNs: A case study of Lake Tisza, Hungary

**DOI:** 10.1007/s10661-025-14662-7

**Published:** 2025-10-24

**Authors:** Ahmed Mohsen, Gábor Fleit, Tímea Kiss, Sándor Baranya

**Affiliations:** 1 Department of Hydraulic and Water Resources Engineering, Faculty of Civil Engineering, Budapest University of Technology and Economics, Műegyetem Rkp. 3, 1111 Budapest, Hungary; 2 Department of Irrigation and Hydraulics Engineering, Tanta University, Tanta, 31512 Egypt; 3 HUN–REN–BME Water Management Research Group, Hungarian Research Network, Műegyetem Rkp. 3, 1111 Budapest, Hungary; 4 National Laboratory for Water Science and Water Safety, Budapest University of Technology and Economics, Műegyetem Rkp. 3, 1111 Budapest, Hungary; 5 Independent Researcher, Horváth Gy. Str. 80, 6630 Mindszent, Hungary

**Keywords:** Suspended sediments, Vegetation coverage, Remote sensing, Spectral indices, Lake Tisza

## Abstract

Shallow reservoirs on large rivers are highly dynamic systems vulnerable to sediment accumulation, eutrophication, and water quality deterioration, posing significant threats to their storage capacity, hydropower generation, and ecological balance. Remote sensing offers a powerful tool for monitoring and enhancing the understanding of suspended sediment dynamics and vegetation distribution. However, many existing studies relied on traditional, less accurate approaches in their analysis. This study aimed to leverage Sentinel-2 imagery (285 images; 2017–2024) and machine learning, specifically artificial neural networks (ANN), to investigate the spatiotemporal distribution of suspended sediment concentration (SSC) and vegetation coverage in the Kisköre Dam reservoir, Tisza River, Hungary. The models showed a promising performance, with the SSC model achieving an R^2^ of 0.87, an MAE of 21.17 g/m^3^, and an RMSE of 22.67 g/m^3^. The land cover classification model achieved an overall accuracy of 0.96. The SSC and vegetation coverage showed a strong association with hydrological, morphological, and meteorological factors alongside the operational regime of the Kisköre Dam. Specifically, high water levels and warmer temperatures in summer were associated with lower SSC and higher vegetation coverage, whereas the opposite condition occurred during low water levels and cold, windy conditions in winter. A downstream decreasing trend in SSC and vegetation extent was observed, highlighting the shallower upstream sub-basins as the most endangered to sedimentation and eutrophication. The findings underscore the potential of remote sensing as a valuable tool for providing critical information on SSC and vegetation data; however, their limitations should also be carefully considered.

## Introduction

Natural lakes and wetlands play a crucial role in conserving biodiversity by providing habitats for various ecosystems (Reynaud & Lanzanova, 2017). The recognition of their environmental, ecological, and economic values has sparked interest in establishing new artificial ecosystems (e.g., reservoirs and wetlands) to support sustainable development and resource management (Mester et al., 2023). Globally, over 50,000 large dams have been constructed, most of which have resulted in the creation of artificial lakes or reservoirs that have significantly transformed landscapes and ecosystems (Lin QiCai, 2011). Despite their numerous benefits, they may also have negative consequences, such as sedimentation, evaporation loss, and communities’ migration (Gibbs & Hickey, 2012).

Dams disconnect sediment transport in rivers through their trapping effect (Mohsen et al., 2023a). Vörösmarty et al. (2003) concluded that 28% of global riverine sediment flux is potentially trapped in reservoirs, amounting to an annual retention of 4–5 billion tons. This trapping not only reduces the storage capacity of reservoirs and decreases hydropower production, but also deprives downstream reaches of essential sediment necessary for sustaining aquatic habitats (Kondolf et al., 2014). Between 1901 and 2010, the storage capacity of large reservoirs worldwide declined by 5% (Wisser et al., 2013). Notably, trapping efficiency rises with longer residence times and higher ratios between reservoir capacity to inflow, as illustrated by the Brune curve (Morris & Fan, 1998). On the other hand, sediment deprivation downstream intensifies the erosion of coastal cliffs and deltas, while also causing the narrowing or disappearance of sediment-starved beaches.

To address sediment trapping challenges, several studies have proposed sediment management strategies that can be broadly classified into three approaches: (1) passing sediment over or around the reservoir, (2) flushing deposited sediment from the reservoir to recover lost capacity, and (3) controlling sediment inflow to the reservoir from upstream reaches (Habersack H. et al., 2019). However, selecting the most appropriate solution requires a holistic understanding of sediment transport and its connections to hydrological, meteorological, and geomorphological conditions, as well as the dam's operational framework. This can be achieved through the effective integration of in-situ data, remote sensing, and numerical modelling techniques. This study addresses limitations of previous research, including limited sample diversity, narrow methodological approaches, lack of contextualization, and weak measurement tools, by integrating extensive multi-year Sentinel-2 data with intensive spatiotemporal in-situ measurements, applying robust ANN-based models that yield more reliable results than simple spectral indices, and explicitly accounting for the ecological context of Lake Tisza.

In the meantime, eutrophication presents an additional environmental challenge, particularly in shallow reservoirs. Excess nutrients, primarily nitrogen (N) and phosphorus (P), from point and non-point pollution sources, as well as recreational activities, are the main drivers accelerating the eutrophication process in aquatic ecosystems (Mester et al., 2023). In addition, climate change-induced droughts and intensive land-use changes contribute to nutrient enrichment (Savic et al., 2022; Woolway et al., 2020). Eutrophication stimulates sedimentation, alters sediment composition, and triggers cascading impacts on reservoir functionality (Li-kun et al., 2017). However, the behavior of eutrophication is strongly influenced by reservoir characteristics, including depth, morphology, residence time, and nutrient inputs (Saito et al., 2013). Therefore, comprehensive vegetation data covering the entire reservoir is vital for tailoring management strategies to each basin.

Lake Tisza in Hungary is an example of an endangered reservoir experiencing sedimentation and vegetation spread. Water caltrop (*Trapa natans*) is the most common vegetation type in the lake (Szabó et al., 2020). Although it is a protected plant and considered vulnerable to extinction in Europe, it poses environmental challenges in the lake by accelerating sedimentation and threatening biodiversity (Hummel & Kiviat, 2004). Remarkably, the proliferation rate of this plant is influenced by water depth, with high intensity in shallow areas (< 2 m) and reduced abundance in deeper areas (Szabó et al., 2020). Similarly, macrophyte patchiness affects hydrodynamics, including changes in near-bed velocity, as well as stimulating sedimentation rates (Folkard, 2005). These changes in hydrodynamics and bathymetry create a feedback loop that further influences macrophyte development (Meire et al., 2014).

Satellite-based remote sensing techniques offer a large-scale, frequent, and cost-effective tool for monitoring spatiotemporal changes in suspended sediment concentration (SSC), as well as vegetation type and density in aquatic ecosystems (Mohsen et al., 2023c). For instance, Chen et al. (2025) used satellite-derived Forel-Ule Index (FUI) data to effectively reveal the spatiotemporal dynamics of water color in 730 lakes across the Yangtze River Basin (1984–2023), along with the climatic and anthropogenic drivers shaping these patterns. However, since the 1970 s, spectral indices have been widely applied for environmental monitoring, including tracking changes in land use/land cover (LULC) and assessing water characteristics in aquatic ecosystems (Ma et al., 2019). However, their accuracy is often limited by sensitivity to atmospheric conditions, the presence of mixed pixels (i.e., pixels containing multiple land cover types), and limited specificity (i.e., difficulty in distinguishing between features with similar spectral characteristics, such as bare soil and urban areas) (Bojinov et al., 2022; Montero et al., 2023).

Recent advancements in machine learning (ML) technology have led to the development of more robust and reliable models, effectively addressing many limitations of traditional approaches. Larson et al. (2021) reported that Landsat 8 machine learning-based SSC models (maximum R^2^ = 0.89) for the Maumee River in Lake Erie outperformed traditional simple linear least-squares regression models. A comparative analysis of four machine learning algorithms, random forest (RF), support vector machine (SVM), decision tree (DT), and artificial neural network (ANN), was conducted to establish a Landsat-based SSC model for Poyang Lake, with the findings indicating the superiority of the RF model, achieving an R^2^ of 0.9 (Liao et al., 2024). A seminal investigation into the application of ANNs for remote sensing-based land cover classification was conducted by Kavzoglu and Mather (2003), who demonstrated the superiority of ANN-based classification over traditional classifiers and proposed practical guidelines for designing effective models. Gao et al. (2024) developed a Sentinel-2 deep learning-based model (i.e., Res-U-Net) incorporating aquatic vegetation data from four lakes along the Yangtze River, China. This model achieved an accuracy of 91% for emergent vegetation, 78% for floating-leaved vegetation, and 59% for submerged vegetation. Similarly, Alagialoglou et al. (2024) used Sentinel-2 images to establish a random forest-based aquatic vegetation model for the Lower Dniester Basin, Ukraine, achieving an F1-score of 0.88 ± 0.03.

Although traditional machine learning algorithms (e.g., RF and SVM) can provide reliable predictions for SSC estimation and land cover classification, this study employed ANNs as they offered more balanced and robust performance in complex aquatic environments. For instance, in estimating SSC in the Lower Tisza River at Szeged, ANNs outperformed RF and SVM (ANN: R^2^ = 0.82; RF: R^2^ = 0.76; SVM: R^2^ = 0.78) (Mohsen et al., 2022). Similarly, ANNs outperformed Decision Trees (DT) and Naïve Bayes (NB) for riverine litter classification in the same river, while delivering results comparable to RF and SVM but with superior generalization capability (Mohsen et al., 2023b). The robustness of ANNs in aquatic and land cover studies has been consistently demonstrated, e.g., (Kavzoglu & Mather, 2003) and (Atkinson & Tatnall, 1997), supporting their adoption here for both SSC and land cover classification models. Their superiority stems from the ability to capture highly nonlinear interactions between spectral bands, which are characteristic of mixed water–vegetation–sediment environments (Mas & Flores, 2008). In contrast, RF and SVM may struggle with subtle nonlinear thresholds when class boundaries overlap. Furthermore, ANNs effectively handle the multicollinearity of satellites’ spectral bands by learning weighted combinations during training, whereas RF may produce redundant splits, and SVM requires careful kernel tuning (Ghayour et al., 2021). In parallel, to address the limitations of the so-called “black-box” nature of ANNs, the Shapley Additive Explanations (SHAP) approach (Lundberg & Lee, 2017) was applied to interpret the contribution of each input feature.

**Fig. 1:**
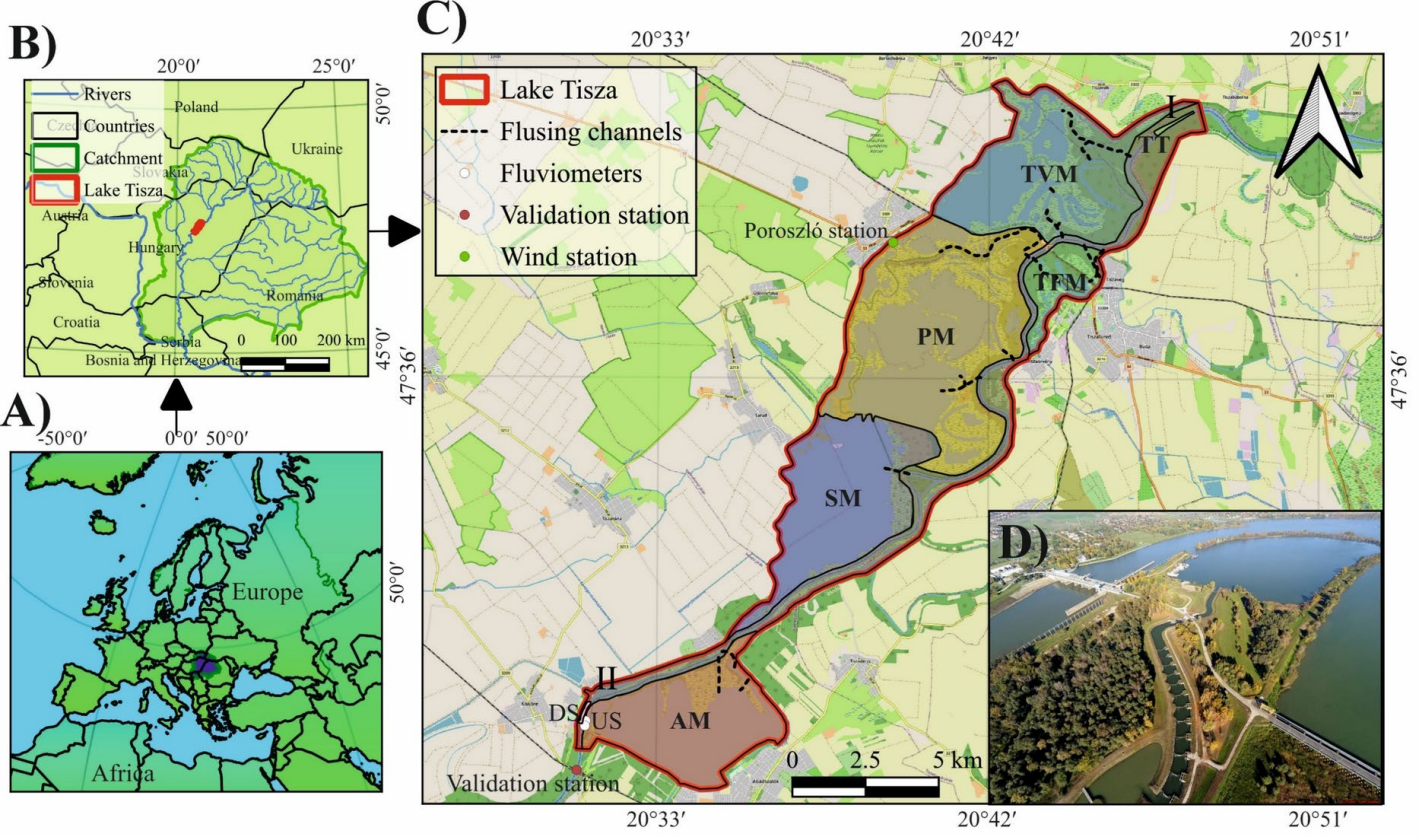
Catchment area of the Tisza River (blue shading with green border) shown in continental (**A**) and national (B; transparent shading with green border) contexts, with emphasis on Lake Tisza and its sub-basins [Tiszavalki Basin (TVM), Poroszlói Basin (PM), Sarudi Basin (SM), Abádszalóki Basin (AM), Tiszafüredi Basin (TFM), and Tisza River basin (TT)] (**C**). Locations of hydrometeorological stations are indicated in panel (**C**), and the position of the Kisköre Dam is shown in panel (**D**). The basemap in panel (**C**) is based on © OpenStreetMap contributors (https://www.openstreetmap.org), while shapefiles from the ArcGIS Hub were used to produce the contextual maps in panels (A) and (B)

Given the elevated sensitivity of Lake Tisza to sedimentation, eutrophication, climate change, and LULC changes, effective conservation plans are essential. The lake is characterized by complex mosaic patterns, including small and shallow basins, large and deep basins, and oxbow channels. These characteristics underscore the need for comprehensive SSC and vegetation data spanning the entire lake across different seasons. This study aims to apply our previously developed ANN-based Sentinel-2 model for the Tisza River (Mohsen et al., 2023c) to investigate the dynamics of SSC in Lake Tisza, and to develop a parallel model for land cover classification in order to examine vegetation coverage dynamics and their interaction with SSC within the lake. The specific objectives are: (1) To explore the correlation between temporal changes in SSC, vegetation coverage, and hydrology, both at the scale of the entire lake and its individual sub-basins (i.e., six distinct hydromorphological units within the larger lake system, separated by natural features, hydrodynamic conditions, and operational plans; Fig. 1) (2) To investigate the influence of dam operational activities on SSC and vegetation coverage. (3) To evaluate the interplay between vegetation coverage and SSC. By achieving these goals, the study will improve our understanding of the sediment and vegetation dynamics in the lake, identify endangered sub-basins, support the selection of the most suitable sediment management strategies, moreover, will provide solid field validation data for numerical simulations. In essence, this study emphasizes the application of well-established remote sensing and machine learning models to reveal the interactions between suspended sediment and vegetation dynamics in shallow lakes, rather than advancing new methodological frameworks.

## Study area

Lake Tisza was artificially formed due to the construction of the Kisköre Dam in the Middle Tisza (400–430 river km) in 1973 to support flood control and generate energy (Fig. 1). It is considered the second largest (five times smaller than Lake Balaton) and the youngest (45 years old) lake in Hungary (Szabó et al., 2020). The lake covers an area of 127 km^2^ (length: 27 km; width: 0.6–6.6 km; mean depth: 1.3 m) and holds a volume of 253 million m^3^ (Mester et al., 2023; Teplán, 2003). The lake is fragmented by several islands (43 km^2^), creating partly isolated sub-basins, while its boundaries are enclosed by flood protection embankments (ICPDR, 2007).

Lake Tisza is located in the Pannonian biogeographical region 2, with a temperate continental climate, characterized by warm to moderately hot summers and cold winters (Metzger et al., 2012). The lake receives relatively low precipitation in the country (mean annual precipitation: < 550 mm) and has large water temperature differences throughout the year (up to 28–30 °C in summer and 0 °C in winter), with an aridity factor of 1.4 (Mester et al., 2023). The prevailing wind direction is from the northeast, although it is occasionally influenced by Mediterranean and Eastern European air masses (Mohsen et al., 2024).

The water stage in Lake Tisza is artificially regulated with the Kisköre Dam to maintain two dominant operational water levels: summer and winter levels. The largest flood waves in the river typically occur in early spring, during which the reservoir is filled (March–April) (Harka et al., 2014). By the end of the filling period, the water stage reaches the summer operational level (725 ± 5, 735 ± 5 cm) and is kept stable until the end of October (Szabó et al., 2020). In November, the water level is lowered by 1.2 m to reach the winter water level. The actual filling and emptying periods last for approximately 2 to 3 weeks. In winter, the water level is maintained at its lowest level (450–610 cm) to ensure flood preparedness and to safeguard infrastructure against ice expansion, as well as for ecological reasons (i.e., to help manage invasive aquatic vegetation) (János, 2019).

Notably, the exchange of water between the river and the reservoir is facilitated by several artificial flushing channels distributed across the various sub-basins. The majority of flushing channels are fitted with locks and are closed when water discharge reaches 800 m^3^/s. This measure prevents sediment-laden floodwaters from entering the reservoir, thereby reducing the risk of sediment deposition and subsequent reservoir sedimentation.

The reported water levels are measured at the “Kisköre upstream” monitoring station, located at the southernmost part of the lake, just upstream of the dam (gauge zero elevation: 81.32 m above mean sea level) (Fig. 1). This water level is considered representative of the entire lake.

Lake Tisza is characterized by complex mosaic patterns of habitations, closely resembling the ancient floodplain of the Tisza River (Harka et al., 2014). It comprises extended water areas, wetlands, paleochannels (oxbows), islands, and large macrophyte-dominated zones, highlighting its significant importance for biodiversity (Szabó et al., 2019). Therefore, the lake was considered a UNESCO World Heritage site, a Ramsar site, and an element of the Natura 2000 Network (Harka, 2014). The land cover of the lake primarily includes floodplain forests, herbaceous and aquatic vegetation, and water (ICPDR, 2018).

Based on the hydro-geomorphological, ecological, and functional criteria of the lake, it is divided into five distinct sub-basins, besides the river itself (Tisza River, TT). Each of these sub-basins has unique operational management plans, including the Tiszavalki Basin (TVM), the Poroszlói Basin (PM), the Sarudi Basin (SM), the Abádszalóki Basin (AM), and the Tiszafüredi Basin (TFM) (Fig. 1).

The TVM is the shallowest sub-basin, with an average water depth of 0.7 m. It is located at the northernmost part of the lake (Fig. 1), and characterized by minimal water flow, near-stationary conditions in certain areas, and an abundance of former natural levees, islands, and channels that support biodiversity (Kókai et al., 2019). Therefore, it is a highly protected sub-basin with limited human interventions. Similar environmental, ecological, and geomorphological settings are present in the PM sub-basin, though it has greater depths (average water depth: 1.3 m). Therefore, it remains a protected area, though some recreational activities, such as kayaking, bird watching, and fishing, are permitted (Mester et al., 2023). The SM sub-basin, with an average water depth of 1.5 m, is located in the middle of the reservoir and is characterized by relatively high and variable water flow. This is due to the influence of both the river and the surrounding sub-basins, resulting in a mix of lentic and lotic flow conditions (Gábor, 2020). The AM sub-basin, characterized by fewer islands and wider and deeper channels (average water depth: 2.5 m) (Mester et al., 2023). As the least protected area, motorized water sports are allowed here. The TFM sub-basin is a former oxbow channel with deep and calm water (max depth: 17 m).

## Material and methods

**Fig. 2:**
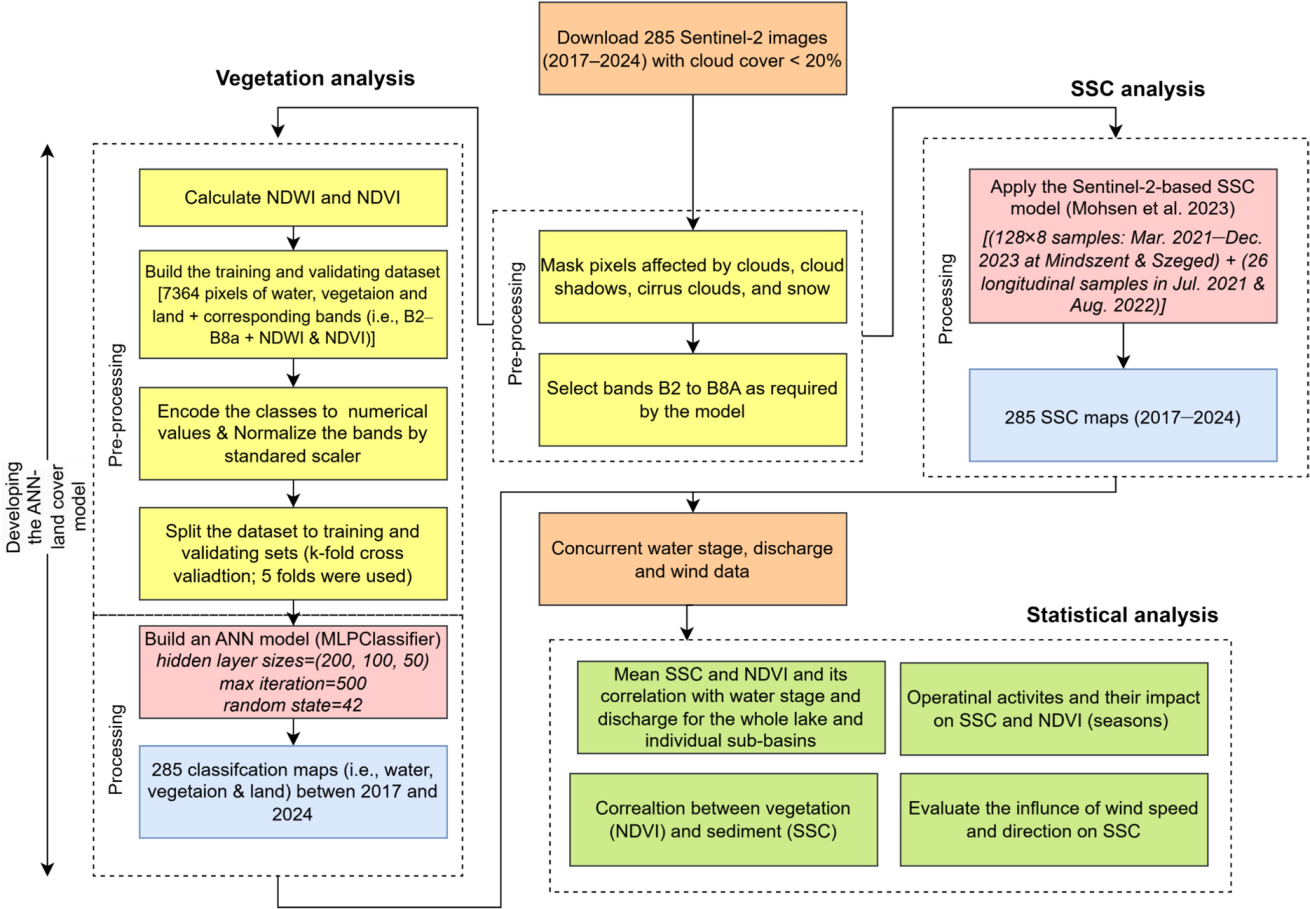
Methodology for investigating the spatiotemporal distribution dynamics of suspended sediment concentration (SSC) and vegetation coverage in Lake Tisza, based on eight years of Sentinel-2 imagery (285 images)

The spatiotemporal distribution dynamics of SSC and vegetation coverage in Lake Tisza were investigated through the effective integration of Sentinel-2 images and machine learning (Fig. 2). The analysis was based on eight years of Sentinel-2 satellite data (285 images) (https://browser.dataspace.copernicus.eu/; accessed on January 12, 2025), along with concurrent water stage, discharge, and in-situ SSC measurements for validation. In addition, an ANN-based land cover model was developed to address the limitations of simple spectral indices. Statistical analysis of mean SSC and NDVI was performed at both the whole-lake scale and for individual sub-basins across seasons, revealing their correlation with hydrological conditions. Additionally, the impact of wind speed and direction on the suspended sediment budget was assessed.

## In-situ data

The hydrological conditions in the lake and the river between April 2017 and December 2024 were characterized using daily water stage and discharge data obtained from the Danube River Basin Hydrological Information System (DanubeHIS) (https://www.danubehis.org/; accessed on January 12, 2025). Water stage was measured both upstream and downstream of the dam (i.e., at Kisköre upstream and downstream, respectively; see Fig. 1), while discharge was recorded only downstream of the dam. In addition, discharge data were collected at the upstream end of the reservoir (Tiszabábolna; Fig. 1), although these data were only available from January 2020 to December 2024. For validation, daily SSC data measured 1.5 km downstream of the dam (Fig. 1) were obtained from the Middle Tisza Hydrological Water Directorate (KÖTIVIZIG). The samples (200–250 ml) were collected from the middle of the channel, near the surface (20 cm depth), using a simple drawstring scoop container and analyzed in the laboratory according to the MSZ 12750–6:1971 standard. The SSC dataset covered the period from April 2017 to December 2023. Additionally, hourly wind speed magnitude (*u*) and direction (u_dir_) measured in the PM sub-basin between 2017 and 2024 were obtained from the Hungarian Meteorological Service (HungaroMet). Although some in-situ measurements were not available for the full study period (2017–2024) (e.g., SSC data, 2017–2023), they were sufficient to validate the satellite-based models. Consequently, our investigation spans from April 2017 to December 2024, relying primarily on satellite-based models and incorporating all available images to ensure more reliable results.

## Remote sensing data

Google Earth Engine (GEE) was used to acquire all available Sentinel-2 images covering Lake Tisza from the “COPERNICUS/S2-SR-HARMONIZED” dataset between April 2017 and December 2024. Only images with less than 20% cloud cover were selected, resulting in a total of 285 images. All images were acquired at Level 2 A from tile 34TDT and relative orbits 36 and 136. Bands B2–B8a were selected, as these were used in the calibration of the SSC model (Mohsen et al., 2023c). To avoid misleading results, pixels with a medium to high probability of cloud cover, cloud shadow, cirrus clouds, or snow were removed. The scene classification layer (SCL) was used to identify and mask these pixels.

## Derivation and validation of suspended sediment concentration (SSC) model

The Sentinel-2-ANN-based SSC model, developed by Mohsen et al. (2023c), was applied to the 285 images to evaluate SSC distribution in Lake Tisza. The model was derived from extensive in-situ SSC measurements in the Tisza River, including 128 samples collected at eight sites in Mindszent and Szeged cities between March 2021 and December 2023, as well as longitudinal measurements from 26 sites along the river, from spring to its confluence with the Danube River, in July 2021 and August 2022. Sentinel-2 bands B2 to B8a were used as independent variables in the SSC model, which was developed using a multilayer perceptron (MLP) feedforward neural network. The model’s hyperparameters were automatically optimized with the Optuna open-source Python library (Optuna n.d.), resulting in three hidden layers (100, 5, and 20 neurons, respectively), a logistic activation function, lbfgs solver, alpha of 0.0003, invscaling learning rate, a maximum of 604 iterations, and a batch size of 67. The model achieved a mean absolute error (MAE) of 23.4 g/m^3^ and an R^2^ of 0.7 over the five folds. The model even achieved better performance on the temporal testing dataset at Mindszent under different hydrological conditions (R^2^ = 0.89), although its performance was lower on the spatial testing dataset in the Middle Tisza (R^2^ = 0.05). Further validation was conducted by comparing the model’s predictions at the validation station (Fig. 1) with concurrent in-situ SSC measurements provided by KÖTIVIZIG (available for 63 images). This model was optimal for our study as it was explicitly developed and validated for the Tisza River (same environment as Lake Tisza), utilising comprehensive in-situ SSC measurements, and showed robust performance under various hydrological conditions.

## Spectral indices calculations

The normalized difference water index (NDWI) (McFeeters, 1996) (Equation A1) and the normalized difference vegetation index (NDVI) (Rouse et al., 1974) (Equation A2) were calculated for all 285 Sentinel-2 images to delineate the lake area and assess vegetation density and coverage within Lake Tisza, respectively. Both indices typically range from −1 to + 1, with water pixels and vegetation generally exhibiting positive values. In particular, higher NDVI values indicate denser and healthier vegetation. For comparative analysis with the findings of Szabó et al. (2020), a fixed threshold of 0 was applied to NDWI images to delineate water areas, which was then used to calculate mean NDVI separately for water and non-water zones. However, since thresholds may vary spatially and temporally, more stringent values were applied when building the land cover model: NDWI and NDVI > 0.4 to identify water and vegetation pixels, and NDVI < –0.4 to identify land pixels. These thresholds were further validated with visual inspection of RGB images to ensure that only pixels with 100% certainty of belonging to each category were included.

## Developing a machine learning-based land cover model

Given the limitations of NDVI, particularly in detecting aquatic vegetation and producing misleading results in complex environments, machine learning algorithms offer a more accurate, adaptable, and scalable alternative. Therefore, an ANN-based land cover model was developed in this study to support numerical modeling by generating dynamic vegetation maps under varying hydro-meteorological conditions.

Altogether 7,364 pixels of water, vegetation, and land were collected for model training and validation. These pixels were manually sampled across different hydro-meteorological conditions, covering almost the entire lake. Besides, to ensure a balanced dataset, each class comprised approximately one-third of the data. The data were sampled every two months in the years of 2017 and 2021. The bands B2–B8a, along with the NDWI and NDVI spectral indices, were used as independent variables in the model. However, to consider the variability of their ranges (i.e., B2–B8a: 0–1; NDWI and NDVI: −1– + 1), a min–max standard scaler was applied to normalize their scales (Equation A3).

The dataset was divided into training and validation sets using k-fold cross-validation, with 5 folds considered. For each fold, (k-1)/k of the data was used for training (80%), while the rest (20%) was used for validation. This approach ensures that every data point is used for both training and validation.

A Multi-Layer Perceptron (MLP) ANN classifier in the scikit-learn library in Python was used to build the land cover model. The ANN is a set of interconnected cells (neurons) arranged in input, hidden(s) and output layers (McCulloch & Pitts, 1943). The weights connecting the neurons are randomly initialized and iteratively updated during training using the backpropagation algorithm to minimize the loss function. In this study, the model included three hidden layers with 200, 100, and 50 neurons, respectively, and used the Rectified Linear Unit (ReLU) activation function. The maximum number of iterations was set to 500, with a random state of 42, the loss function set to “binary cross-entropy”, and the optimizer set to “adam”.

## Statistical analysis

To investigate the spatiotemporal distribution dynamics of suspended sediment and vegetation in the lake: (1) The mean SSC and NDVI for the whole lake were calculated and compared with the water stage upstream and discharge downstream of the dam. (2) The mean SSC and NDVI for the whole lake were calculated across four operational periods of the dam, corresponding to the four seasons (i.e., spring filling, summer high water stage, autumn emptying, and winter low water stage). (3) The mean SSC and NDVI were compared across the six sub-basins and during the four seasons. (4) The correlation between sediment (in terms of SSC) and vegetation (in terms of NDVI) was evaluated considering the whole lake. (5) Wind speed and direction were compared to SSC throughout the entire study period, as well as across different seasons.

Notably, the mean NDVI was calculated not only for the entire lake “water + non-water” but also separately for land and water to account for differences in vegetation coverage. Specifically, pixels with an NDWI greater than 0 were classified as "water," while pixels with an NDWI of 0 or less were classified as "non-water" (land). This classification was necessary to clearly separate the two classes and to calculate the mean NDVI for each class individually. A fixed NDWI threshold of 0 was applied in this study, both for comparability with the results of Szabó et al. (2020), who used the same threshold, and because it aligns with many observed cases in Lake Tisza (Figure A9). Nonetheless, it should be acknowledged that the NDWI threshold can vary spatially and temporally, particularly in shallow lakes. Addressing this dynamic variability, however, is beyond the scope of the present study.

Since the Sentinel-2 images were acquired at 09:40 UTC, wind data from 08:40 UTC were paired with the SSC measurements to account for the physical lag and process timing in sediment dynamics driven by wind. This approach provides a more comprehensive representation of wind effects than using daily mean values. Additionally, the continuous hourly wind dataset spanning the study period (2017–2024) was used to generate a wind rose.

The classification criteria of the Pearson correlation coefficient (r) proposed by Akoglu (2018) were applied to describe the strength of correlations between parameters. Specifically, correlations were categorized as very weak (0.0 ≤ r ≤ 0.19), weak (0.2 ≤ r ≤ 0.39), moderate (0.4 ≤ r ≤ 0.59), strong (0.6 ≤ r ≤ 0.79), and very strong (0.8 ≤ r ≤ 1.0).

## Results

### Validation of the satellite-based suspended sediment concentration estimates

**Fig. 3:**
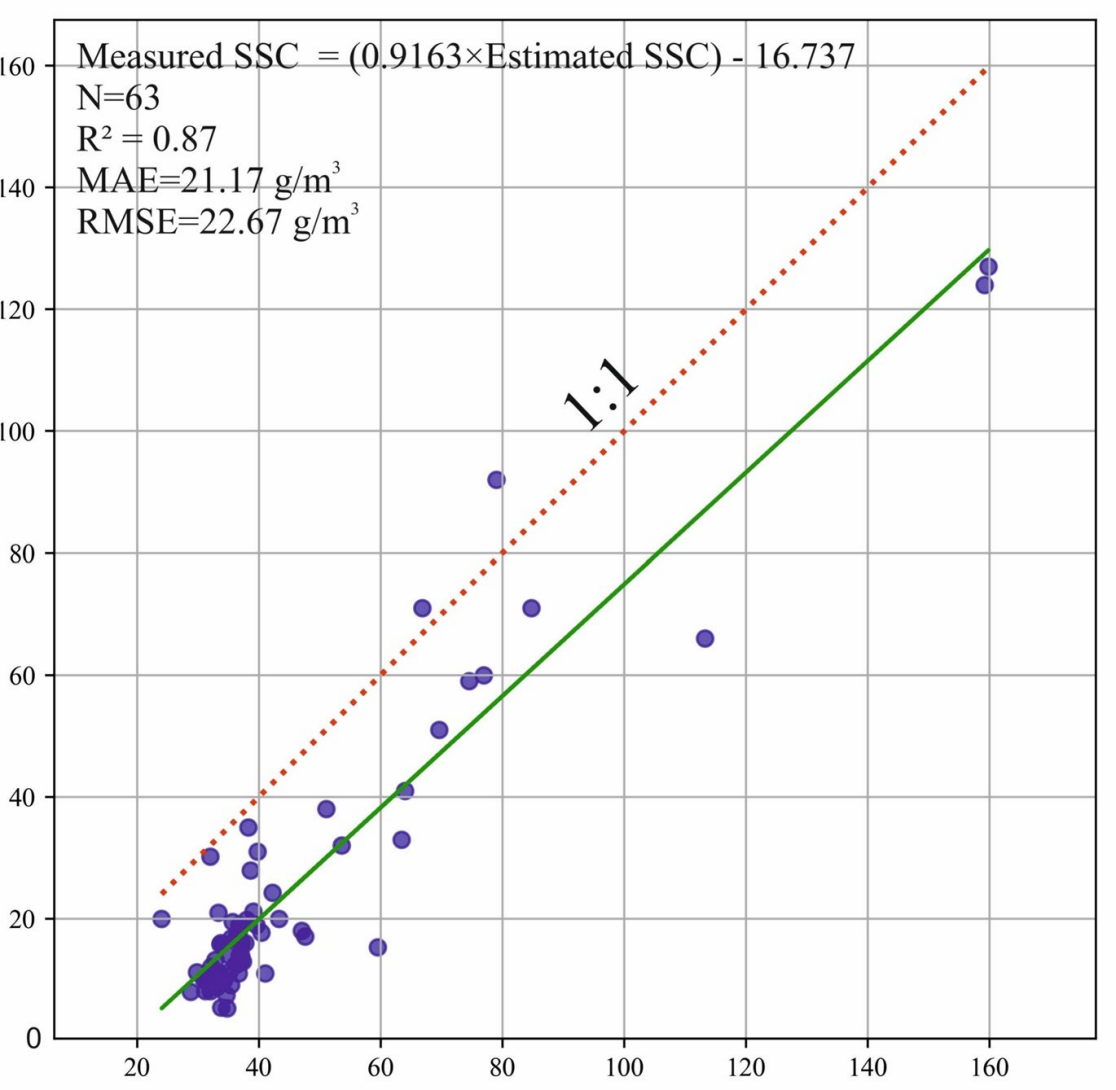
Scatter plot between measured and satellite-based estimated suspended sediment concentration (SSC) at the validation site across 63 concurrent dates (2017–2023)

The comparison between measured and satellite-based SSC estimates at the validation site (63 concurrent dates) revealed strong agreement, with an R^2^ of 0.87, an MAE of 21.17 g/m^3^, and an RMSE of 22.67 g/m^3^ (Fig. 3 and Figure A1). However, the satellite-based model consistently overestimated the measured SSC values, likely due to the differing scope of the in-situ measurements, which focused on suspended sediment in the upper water layer (approximately 20 cm depth), as opposed to the true surface SSC observed by satellites. Therefore, a bias correction was applied using the linear fit from Fig. 3 to calibrate the SSC estimates. In the meantime, it is important to note that this model was not fitted to the local dataset of Lake Tisza. Instead, it was based on long-term spatiotemporal SSC data measured along the river and at specific sites. The close agreement with the local data, therefore, indicates the model's robustness and reliability.

## Temporal change of suspended sediment concentration in the lake in relation to hydrology

**Fig. 4:**
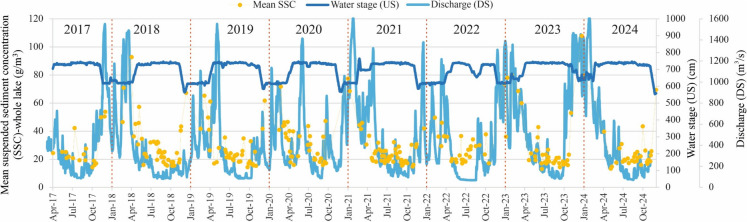
Timeseries of mean suspended sediment concentration (SSC) in Lake Tisza (2017–2024) compared to water stage upstream and discharge downstream of the dam

Throughout the study period (2017–2024), the mean SSC in Lake Tisza was 28.3 ± 14.9 g/m^3^ (Fig. 4). The lowest concentration, 13.1 g/m^3^, was recorded on June 20, 2023, while the highest concentration, 107.8 g/m^3^, was recorded on December 22, 2023. A strong negative correlation was observed between the mean SSC in the lake and the upstream water stage (r = −0.71) (Figure A2A), whereas a moderate positive correlation was found with the downstream discharge (r = 0.53) (Figure A2B). This indicates that SSC is lowest during high upstream water stages, between April and October in each year, and highest during low stages, between November and March, while the opposite trend occurs with the downstream discharge. Notably, the mean SSC in the lake showed a stronger association with the upstream water stage than with the downstream discharge (Fig. 4 and Figure A2). The positive correlation with flow discharge can be explained by the higher sediment transport capacity of the flow. In contrast, the negative correlation with water levels reflects either the impact of reservoir operation or the distinct meteorological conditions between summer, i.e., high water level, and winter, i.e., low water level, as the water level remains relatively steady during the two periods. Notably, the highest SSC in the lake typically precedes the discharge peak observed at the downstream river site, indicating a potential clockwise hysteretic pattern (i.e., a looped relationship between SSC and Q, where SSC is higher on the rising limb than on the falling limb at the same discharge, reflecting an initial surplus of sediment supply at the onset of the flood wave and its rapid depletion thereafter).

## Seasonal change of suspended sediment concentration in the lake (operational activity of the dam)

Considering seasonal variations in mean SSC during the study period (2017–2024), Lake Tisza showed the lowest concentration during high water levels in summer (22.7 ± 6.5 g/m^3^) and the highest concentration during low water levels in winter (60.9 ± 18.4 g/m^3^) (Figure A3). Moderate concentrations occurred in spring and autumn, with spring SSC (34.6 ± 15.7 g/m^3^) being 1.2 times higher than in autumn (28.2 ± 12.5 g/m^3^). As already mentioned before, the mean SSC revealed a negative correlation with the upstream water stage and a positive correlation with the downstream discharge.

**Fig. 5:**
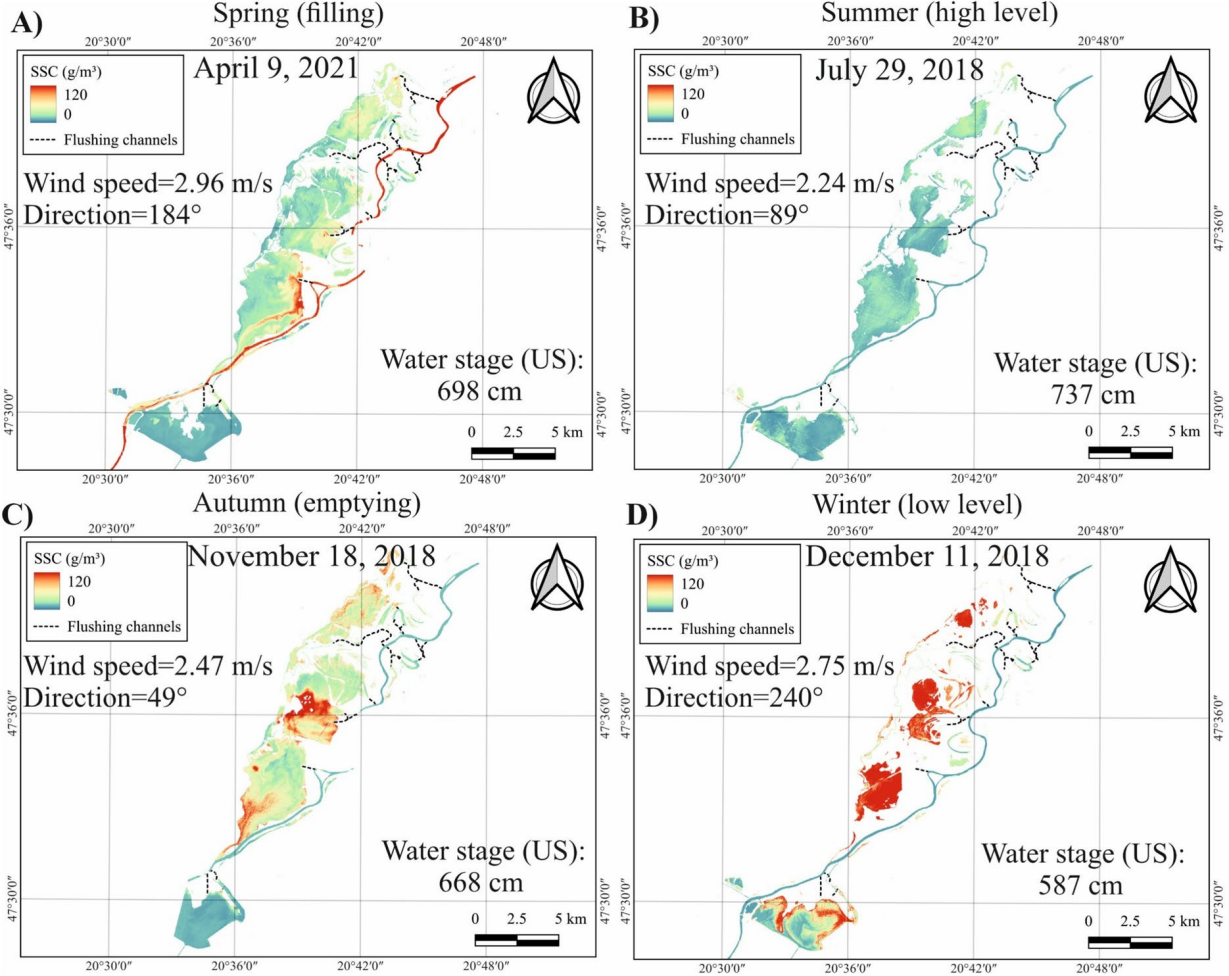
Examples of suspended sediment concentration (SSC) patterns on selected dates represent the influence of four operational periods (i.e., spring-filling, summer-high level, autumn-emptying, and winter-low level) on sediment concentration in the river and the lake

The seasonal spatial distribution of SSC was depicted through Sentinel-2 images acquired on specific dates across different seasons (Fig. 5). To minimize the potential influence of wind-induced resuspension, comparable calm days were selected across seasons for a more reliable comparison. In spring (April 9, 2021), SSC was typically higher in the river than in the lake due to flood waves (Fig. 5A). During this period, the flushing channels were still open, that is, sediment-laden water is allowed to enter the lake. Consequently, high SSC can be observed near the flushing channel outlet zone. Remarkably, SSC in the river decreased toward the dam, but a sudden increase occurred just downstream of the dam. In summer (July 29, 2018), both the river and the lake showed low SSC, although the mean concentration in the lake was slightly higher than in the river (Fig. 5B). No significant variations in SSC are noticed across the lake's sub-basins. In Autumn (November 18, 2018), the flushing channels are opened to maintain ecological balance and increase storage capacity for upcoming flood waves. This process results in higher SSC in the lake than in the river (Fig. 5C). Significantly lower SSC was noticed in the AM sub-basin compared to the other sub-basins, especially the SM sub-basin. A similar pattern is observed during low levels in winter (December 11, 2018), though the SSC difference between the river and the lake, as well as among the sub-basins, is significantly more pronounced (Fig. 5D).

## Suspended sediment concentration in the lake`s sub-basins

**Fig. 6:**
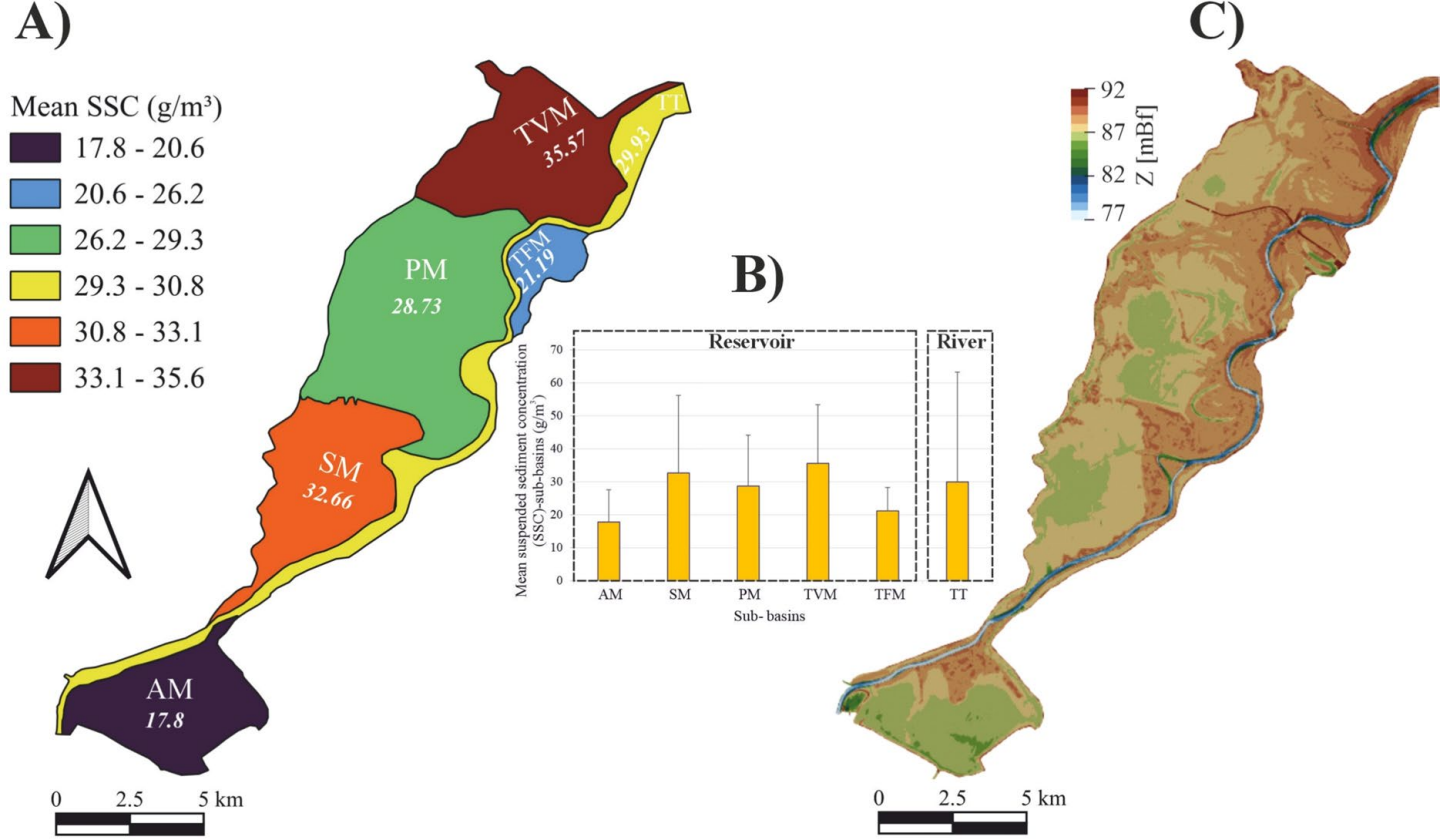
Mean suspended sediment concentration (SSC) across the six sub-basins based on 285 Sentinel-2 images (2017–2024) (A and B). Digital terrain model (DTM) of the lake derived from recent acoustic doppler current profiler (ADCP) depth measurements (C)

The spatial variability of SSC across the six sub-basins of the lake was analyzed and depicted (Fig. 6A and B). Notably, the highest SSC was observed in the uppermost TVM sub-basin (mean = 35.6 ± 17.8 g/m^3^), while the lowest concentration was recorded in the lowermost AM sub-basin (mean = 17.8 ± 9.8 g/m^3^). A general decreasing trend was noticed from the upper to the lower sub-basins, though only a slight decline was noted in the PM sub-basin (mean = 28.7 ± 15.4 g/m^3^). A relatively low concentration occurred in the TFM sub-basin (mean = 21.2 ± 7.1 g/m^3^), while a moderate concentration was observed in the river (i.e., TT sub-basin) (mean = 29.9 ± 33.3 g/m^3^).

The Pearson correlation matrix between mean SSC in the six sub-basins and the water stage revealed a negative correlation with the upstream water stage and a positive correlation with the downstream discharge, with a stronger correlation observed at the upstream stage (Table 1). Focusing on the individual sub-basins, the shallow sub-basins (i.e., TVM, PM, SM) showed a stronger correlation with water stage upstream (r = −0.67–−0.72) compared to the deeper sub-basins (i.e., AM, TFM, and TT) (r = −0.29–−0.37). The downstream discharge exhibited the strongest correlation with the TT sub-basin (i.e., the river itself), with a correlation coefficient of r = 0.76.

**Table 1:** The Pearson correlation coefficients (r) between water stage (upstream), discharge (downstream), and mean suspended sediment concentration (SSC) in the six sub-basins of the lake

		Water stage	Discharge		Sub-basins					
		Upstream		Downstream	AM	SM	PM	TVM	TFM	TT
**Water stage**	Upstream	1								
**Discharge**	Downstream	−0.36		1						
**Sub-basins**	AM	−0.37		0.21	1					
	SM	**−0.68**		0.38	0.51	1				
	PM	**−0.67**		0.32	**0.59**	**0.9**	1			
	TVM	**−0.72**		0.38	0.47	0.78	**0.81**	1		
	TFM	−0.29		0.22	0.32	0.30	0.37	**0.57**	1	
	TT	−0.30		**0.76**	0.14	**0.30**	0.23	0.28	0.26	1

Concerning correlations among SSC across the six sub-basins, the AM, SM, and TVM sub-basins showed the strongest correlation with the PM sub-basin, given its transitional nature, shifting from upstream shallow, highly protected sub-basins to deeper, less protected downstream sub-basins. The TT sub-basin showed its highest correlation with the SM sub-basin (r = 0.3), compared to other sub-basins (r = 0.14–0.28), suggesting strong hydraulic connectivity, likely due to its short and direct flushing channel compared to other sub-basins. Finally, the TFM sub-basin revealed the strongest association with the TVM sub-basin (r = 0.57), compared to other sub-basins (r = 0.26–0.37), due to their close proximity and sharing several morphological and ecological characteristics.

**Fig. 7:**
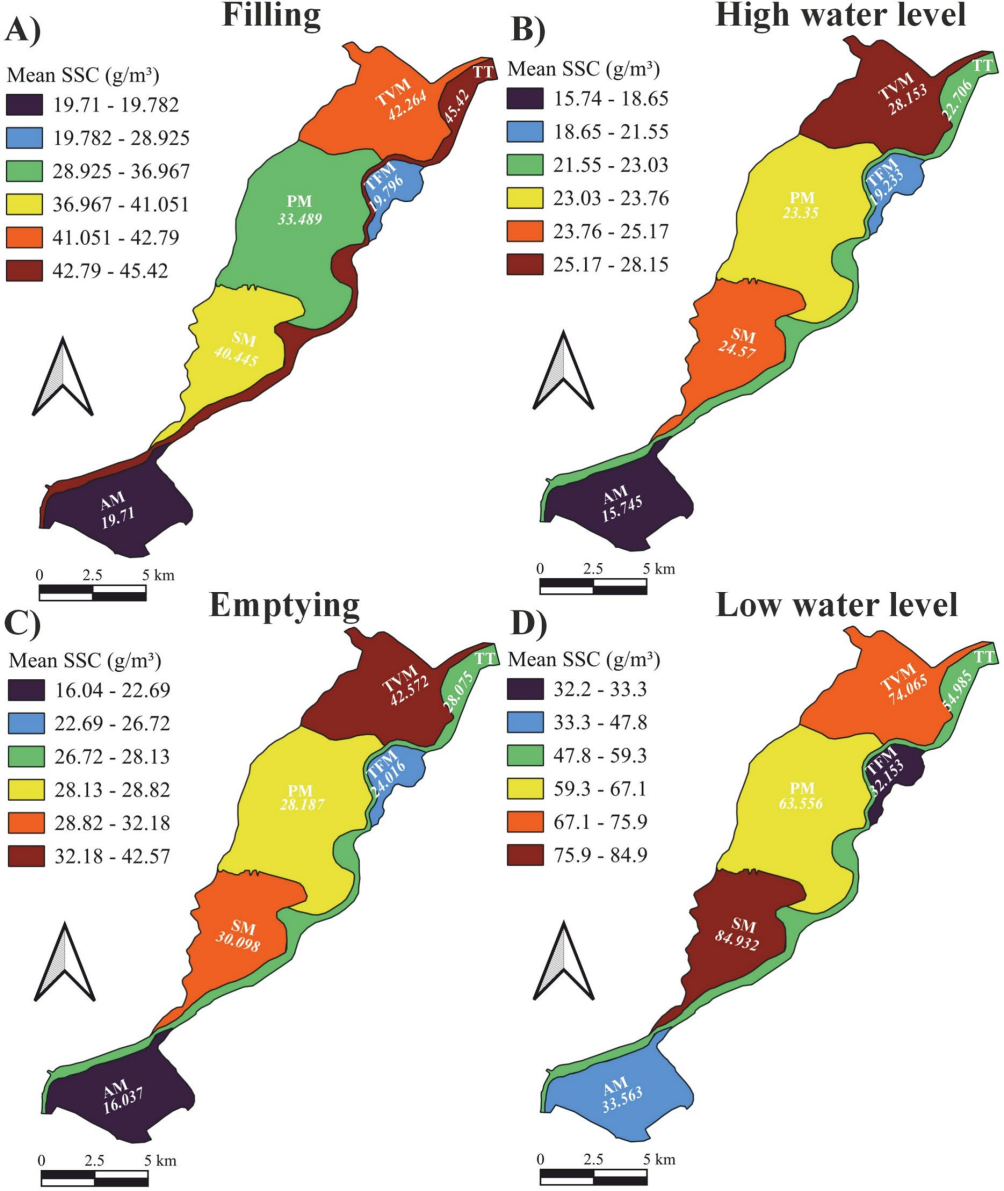
Seasonal variations in mean suspended sediment concentration (SSC) across the six sub-basins of Lake Tisza. The data are based on the analysis of 285 Sentinel-2 images between 2017 and 2024

The seasonal variations in mean SSC in the six sub-basins were calculated and depicted (Fig. 7). The typical spatial SSC pattern identified in Fig. 6A and B was also noticed in spring (i.e., filling), summer (i.e., high water level), and autumn (i.e., emptying). Meanwhile, in winter (i.e., low water level), the highest SSC was recorded in the SM sub-basin instead of the TVM sub-basin, while the rest of the spatial distribution remained unchanged. Focusing on the individual sub-basins and comparing seasonal SSC variations with the typical pattern identified in Figure A3, all sub-basins followed the same temporal trend, except for the TFM sub-basin, where the mean SSC in autumn was slightly higher than in spring.

## Evaluation of the developed land cover model

The developed land cover model showed promising classification results, with the accuracy, precision, recall, and f1-score all exceeding 0.94 across the five folds (Figure A4A). Based on the confusion matrix, the model showed a notable performance with water, correctly classifying all 558 testing pixels (Figure A4B). The vast majority (459) of the vegetation testing pixels were correctly classified, with false negative pixels being equally distributed between land (2 pixels) and water (2 pixels). Similarly, most of the land testing pixels (445) were correctly classified, with false negatives occurring only with vegetation (6 pixels).

## Temporal change of the vegetation coverage in the lake in relation to hydrology

**Fig. 8:**
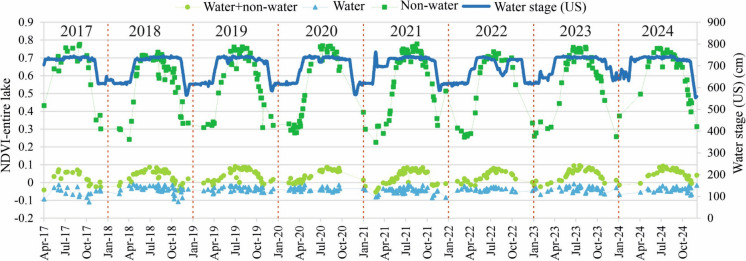
Timeseries of the mean normalized difference vegetation index (NDVI) for the entire Lake Tisza, considering three areas— “water + non-water”, “water”, and “non-water”. The data are based on the analysis of 285 Sentinel-2 images between 2017 and 2024

The magnitude of the NDVI for the “non-water” area was the highest (mean: 0.58 ± 0.16), followed by the “water + non-water” area (mean: 0.04 ± 0.03), while the “water” area showed the lowest NDVI (mean: −0.04 ± 0.02) (Fig. 8). Notably, the highest vegetation coverage occurred during high water stages (i.e., summer and spring) and the lowest occurred during low stages (i.e., winter and autumn). The magnitude of this seasonal variability was more pronounced in “non-water” areas, followed by “water + non-water” areas, while it remained almost stable in “water” areas. This was also inferred from the correlation analysis between water stage and NDVI, which revealed a strong correlation for “non-water” areas (r = 0.78), a moderate correlation for “water + non-water” areas (r = 0.55), and a very weak correlation for “water” areas (r = 0.06) (Figure A5D). Remarkably, the regression pattern was well represented with an exponential equation, especially in “non-water” and “water + non-water” areas (Figure A5C and A), indicating subtle changes in vegetation coverage during the low stage, with a sharp increase occurring during the high stages.

## Seasonal change of vegetation coverage in the lake (operational activity of the dam)

The seasonal analysis of the vegetation coverage in the lake, considering the “non-water” area, revealed the highest coverage in summer (mean NDVI: 0.68 ± 0.12), and the lowest in winter (mean NDVI: 0.32 ± 0.06) (Figure A6). The coverage in spring (mean NDVI: 0.407 ± 0.06) and autumn (mean NDVI: 0.412 ± 0.06) was comparable. For the “water + non-water” area, the highest vegetation coverage was also observed in summer (mean NDVI: 0.05 ± 0.02), while the lowest occurred in autumn (mean NDVI: −0.009 ± 0.019). For the “water” area, all NDVI values across the seasons were negative, indicating no vegetation, though the highest magnitude occurred in spring (mean NDVI: −0.039 ± 0.021) and the lowest in autumn (mean NDVI: −0.054 ± 0.016).

**Fig. 9:**
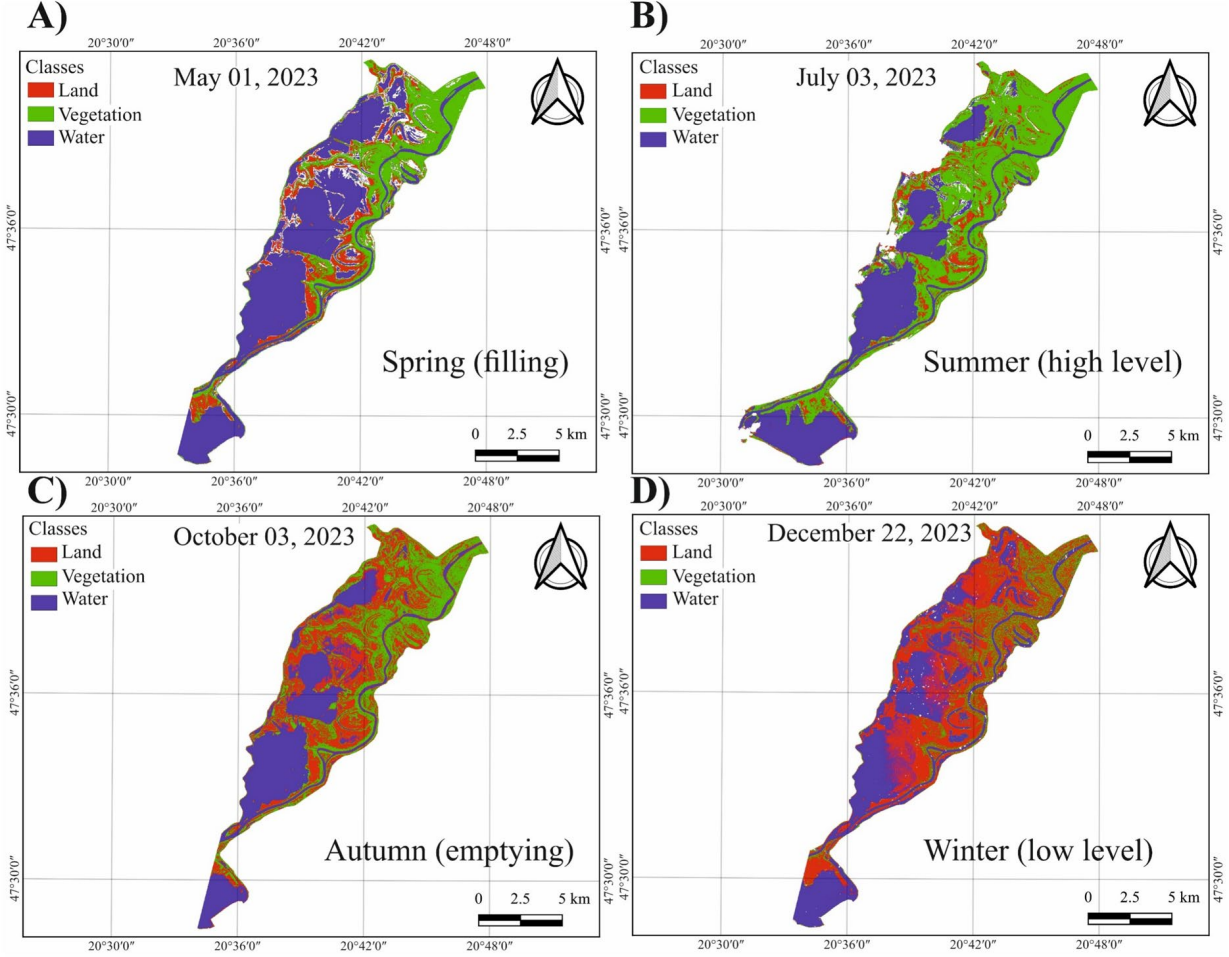
Seasonal variability of vegetation coverage in Lake Tisza. The maps are based on the application of the developed artificial neural network (ANN)-based land cover model to Sentinel-2 images acquired on May 01, 2023 (A), July 03, 2023 (B), October 03, 2023 (C), and December 22, 2023 (D)

The developed ANN-based land cover model was applied to selected Sentinel-2 images acquired in different seasons of 2023 (i.e., May 01st, July 3rd, October 10th, and December 22nd) (Fig. 9). A gradual increase in vegetation coverage occurred during spring, reaching its peak in summer, then declining again in autumn until reaching the lowest values in winter. This distribution closely aligns with reservoir operational activities, resulting in the highest water levels in summer and the lowest in winter. Throughout the seasons, the highest and most persistent vegetation coverage is typically found along the river floodplain. Within the lake, vegetation coverage is the highest in the uppermost protected area and decreases downstream.

## *Vegetation* coverage *in the lake`s sub-basins*

**Fig. 10:**
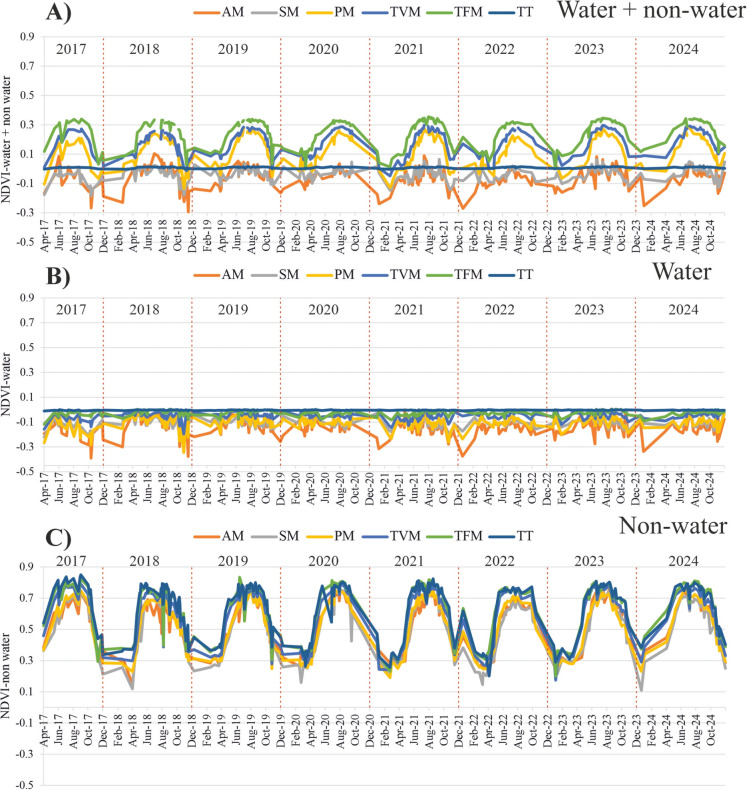
Timeseries of the normalized difference vegetation index (NDVI) for the six sub-basins of Lake Tisza, considering “water + non-water” (A), “water” (B), and “non-water” (C) areas. The data are based on the analysis of 285 Sentinel-2 images between 2017 and 2024

Considering the “water + non-water” area, it can be noticed that vegetation coverage is the highest in the TFM sub-basin (mean NDVI: 0.25 ± 0.09), gradually declining until reaching the lowest values in the AM sub-basin (mean NDVI: −0.05 ± 0.07) (Fig. 10A and Figure A7). Vegetation seasonality was most pronounced in the TFM, TVM, and PM sub-basins, declining in the AM and SM sub-basins, and remaining almost stable in the TT sub-basin. For the “water” area, almost all sub-basins indicated negative NDVI values, with the highest values in the TT sub-basin (mean NDVI: −0.004 ± 0.002) and the lowest in the AM sub-basin (mean NDVI: −0.135 ± 0.067) (Fig. 10B and Figure A7). Considering the “non-water” area, vegetation coverage is the highest in the TT sub-basin (mean NDVI: 0.65 ± 0.16), gradually declining downstream to the lowest value in the AM sub-basin (mean NDVI: 0.55 ± 0.15) (Fig. 10C and Figure A7). For this area, seasonality was significant and consistent across all sub-basins.

**Fig. 11:**
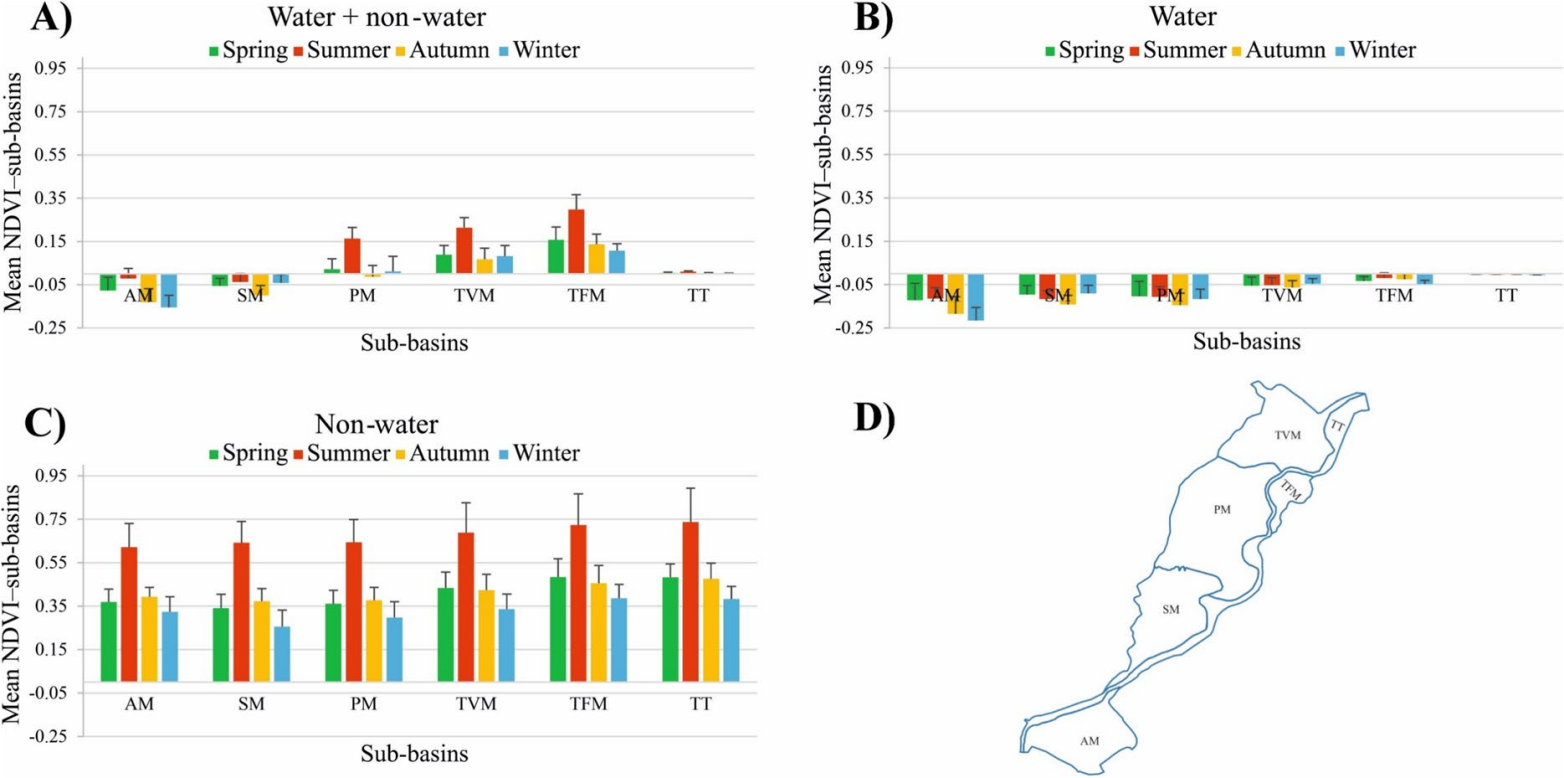
Seasonal variability of the mean normalized difference vegetation index (NDVI) in the six sub-basins of Lake Tisza, considering “water + non-water” (A), “water” (B), and “non-water” (C) areas. The data are based on the analysis of 285 Sentinel-2 images between 2017 and 2024. The lake sub-basin layout is presented in Panel D

Almost all sub-basins follow the typical seasonal vegetation pattern identified in Figure A6, with the highest coverage in summer and the lowest values in winter, particularly for the “non-water” area (Fig. 11). However, slight deviations from the typical pattern were observed in certain sub-basins for “water” areas (Fig. 11B). Comparing the vegetation coverage across sub-basins in each season, it can be noticed that all sub-basins followed the same typical pattern identified in Figure A7 for each area (i.e., “water + non-water”, “water”, and “non-water” areas).

## The influence of vegetation on suspended sediment concentration in the lake

**Fig. 12:**
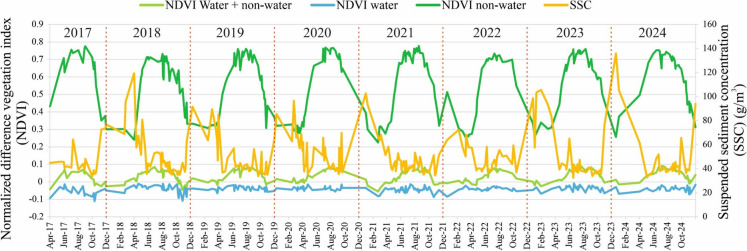
Timeseries of the normalized difference vegetation index (NDVI) for different areas (i.e., “water + non-water”, “water”, and “non-water”), in relation to suspended sediment concentration (SSC). The data are based on the analysis of 285 Sentinel-2 images between 2017 and 2024

The temporal change in SSC in the lake followed an opposite pattern to vegetation coverage, where the highest concentrations occurred during low vegetation coverage in winter and the lowest concentrations occurred during high vegetation coverage in summer (Fig. 12). Therefore, the vegetation peak typically lags behind the corresponding SSC peak. This pattern was typical in the “water + non-water” and “non-water” areas, where a weak (r = −0.37) to moderate (r = −0.58) negative correlation was found between SSC and NDVI, respectively. In contrast, a very weak correlation was observed between SSC and NDVI in the ‘water’ area (r = 0.04), where vegetation exhibited minimal seasonality.

The relationship between SSC and vegetation dynamics in Lake Tisza reflects the coupled influence of hydrology, sediment transport, and riparian productivity. The negative correlation between SSC and NDVI in non-water areas suggests that the availability of water and solar radiation during summer promotes vegetation growth, while simultaneously diluting suspended sediments and leading to reduced concentrations. Conversely, in winter, low water level and reduced solar radiation constrain vegetation development, whereas elevated turbulence from higher wind speeds contributes to increased SSC levels. In contrast, the very weak correlation between SSC and NDVI in water areas is expected, as open water surfaces exhibit minimal photosynthetic activity and weak seasonal NDVI variability. Nevertheless, further research focusing on aquatic vegetation and its interaction with SSC is warranted, ideally through the integration of higher-resolution satellite data and in-situ observations.

## Discussion

After validating the ANN-based SSC model (Mohsen et al., 2023c), it was employed to investigate the spatiotemporal distribution dynamics of SSC in Lake Tisza using a relatively large dataset of Sentinel-2 images (i.e., 285 images; 2017–2024). In addition, an ANN-based land cover model was developed to investigate the spatial and temporal variability in vegetation coverage across the lake. The study revealed the influence of hydro-meteorological conditions and the dam's operational scheme on both SSC and vegetation coverage across different seasons and sub-basins. Furthermore, the interplay between vegetation coverage and SSC distribution was assessed.

## Temporal variations of suspended sediment concentration and vegetation coverage in Lake Tisza

Based on the analysis of eight years of Sentinel-2 images (2017–2024), the mean SSC in Lake Tisza (28.3 ± 14.9 g/m^3^) was close to the reported value (20 g/m^3^) by Kéri et al. (2021). Meanwhile, the identified SSC range in Lake Tisza (13.1 g/m^3^–107.8 g/m^3^) was lower than in Lake Balaton, Hungary (50–150 g/m^3^) (Luettich Jr et al., 1990). The mean SSC in the lake revealed a stronger correlation with the upstream water stage than with the downstream discharge, as the upstream site is more representative of the lake`s water level, while the downstream site corresponds more to the river`s hydrology, aligning with Zhang et al. (2025) in the Liaodong Bay, China. Although SSC showed a negative correlation with the upstream water stage, vegetation coverage revealed a positive correlation. This is likely due to the dilution effect and sediment deposition during high water stages, and the favorable conditions for vegetation growth (Xu et al., 2022). Conversely, during low water stages, increased turbulence from wind action resuspends deposited sediments, while the reduced water volume further concentrates them, leading to higher SSC, as reported for Lake Okeechobee, a large shallow lake in Florida, U.S.A. (Sheng et al., 1995). At the same time, drought stress may further reduce vegetation coverage.

The analysis revealed a potential clockwise hysteresis in SSC, with the lake’s SSC peak occurring before the river’s discharge peak (Fig. 4). A similar pattern was also observed in the Lower Tisza River at Szeged, based on in-situ SSC data collected between 2015 and 2020 (Mohsen et al., 2022). This hysteretic pattern in the lake can be attributed to its hydrodynamic characteristics and the dam’s operational regime, which introduce a time lag between SSC and discharge peaks. In other words, turbulence during low water stages and decreased water volume elevate SSC; meanwhile, as the flood progresses, high discharge promotes sediment deposition, reducing SSC (Szabó et al., 2020). On the other hand, vegetation coverage reached its peak in summer, after the discharge peak, with a marked and rapid increase observed during spring and early summer. This pattern is likely because of the interplay of several factors conducive to vegetation growth during this period, including warmer temperatures (Figure A8), increased water availability, longer daylight hours, and nutrient-rich runoff during flood waves (Coffey et al., 2018).

It should be noted that most of the SSC data used in this study were collected under low to moderate discharge conditions (Fig. 4), largely because optical satellite imagery is frequently unavailable during floods due to cloud cover. Although flood periods are relatively short compared to low-flow conditions, the proportion of SSC data available during floods remains lower. Therefore, the present results are most reliable under typical hydrological conditions and should be interpreted with caution when extrapolated to extreme flood scenarios. Future studies that integrate complementary data sources and targeted in-situ measurements during high-flow events are recommended to better capture SSC dynamics across the full hydrological spectrum.

The mean NDVI in the lake was the highest in “non-water” areas (0.58 ± 0.16), followed by “water + and non-water” areas (0.04 ± 0.03), with the lowest values observed in “water” areas (0.04 ± 0.02), aligning with findings by Szabó et al. (2020). A similar pattern was observed in vegetation seasonality, where the most pronounced seasonal variation occurred in “non-water” areas and gradually declined toward “water” areas. This is primarily due to the presence of chlorophyll in vegetation (i.e., “non-water” areas) and its absence in water (i.e., “water” areas). Since chlorophyll drives photosynthesis activities, its seasonal variability leads to elevated fluctuations in NDVI across seasons (Yang et al., 2017).

The seasonal analysis revealed that the lowest SSC occurs in summer due to high water levels and consequently low mixing and sedimentation. In contrast, the highest SSC occurred in winter, likely due to reduced dilution (low water levels), increased wind action, and wave-induced turbulence, all of which contribute to elevated SSC. In the East China Seas, seasonal retrievals using SeaWiFS imagery showed that SSC are remarkably higher in winter than in summer, even within the same location, consistent with our observations in Lake Tisza (Wang & Jiang, 2008). However, it should be noted that the very shallow water in winter may also influence the recorded reflectance and, consequently, the estimated SSC due to the interference from bottom lakebed reflectance (Ma et al., 2021). Moderate concentrations were observed in spring and autumn, though slightly higher concentrations occurred in spring. This suggests that sediment input from spring floods has a slightly greater impact on SSC than sediment resuspension caused by water level fluctuations during reservoir emptying in autumn. Remarkably, these seasonal changes not only led to discrepancies in SSC between the lake and the river but also among the lake`s sub-basins (Fig. 5).

The seasonal variation in vegetation coverage showed an adverse pattern to that of SSC, with the highest coverage occurring in summer and the lowest in winter, while spring and autumn had comparable values (Figure A6). Since this trend was observed in “non-water” areas, it is better explained by seasonal changes in meteorological conditions and water levels rather than direct interactions between vegetation and sediment. Specifically, warmer temperatures and sufficient water availability in summer stimulate vegetation growth, while unfavourable winter conditions deter it. This pattern aligns with the typical vegetation dynamics in temperate regions like Hungary (Vass & Túri, 2021). The mean NDVI in “water” areas revealed minimal seasonal variability, likely due to the low percentage of aquatic vegetation, resulting in water pixels dominating the mean NDVI for this class, consistent with Szabó et al. (2020).

**Fig. 13:**
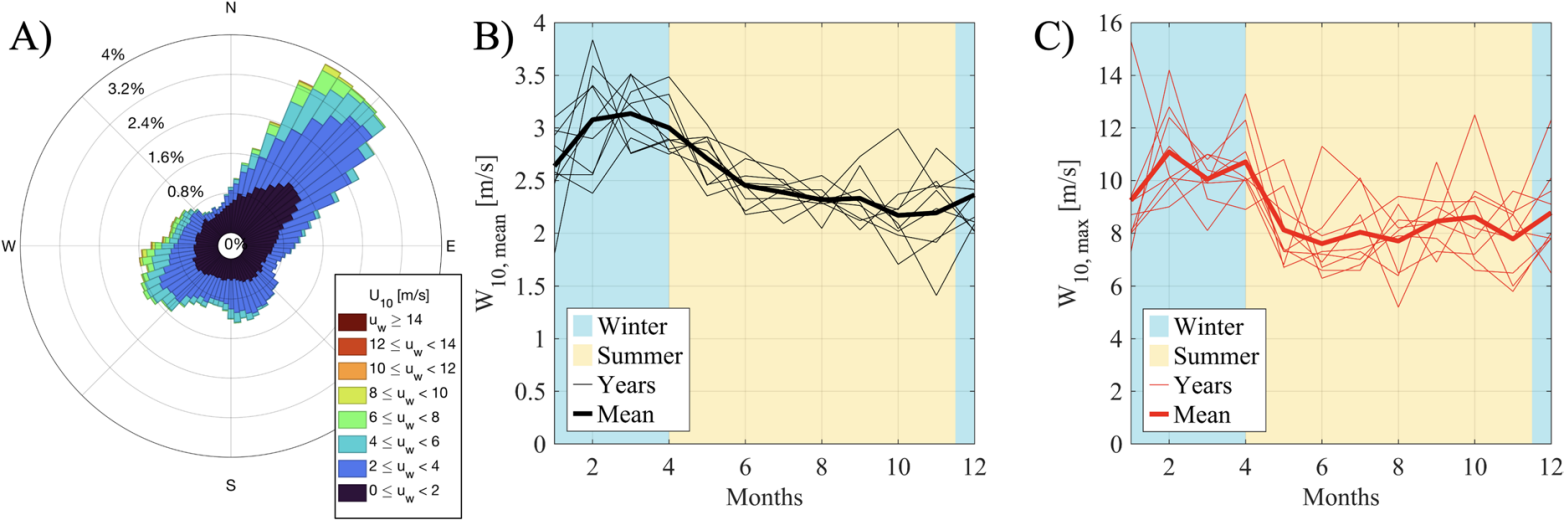
Wind rose for Lake Tisza based on hourly wind speed and direction between 2013 and 2024 (A) and monthly mean (B), and maximal (C) for each year

Lake Tisza generally experiences light winds, with an average speed of 2.51 ± 1.56 m/s, predominantly blowing from the northeast (prevailing wind direction: 43°). However, very strong winds exceeding 14 m/s can occur from most directions, as illustrated in Fig. 13, highlighting a clear seasonal pattern in wind speeds, with stronger winds typically occurring in winter and lighter winds in summer. This seasonal variation corresponds closely with dam operations, specifically the differing water levels in summer and winter.

**Fig. 14:**
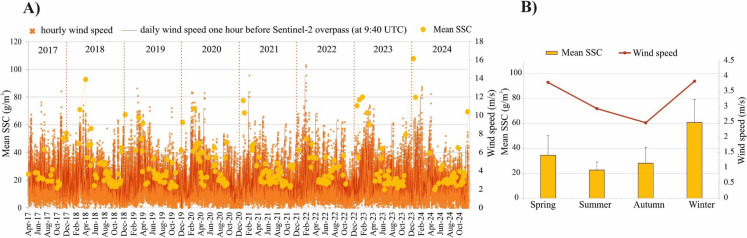
Timeseries of wind speed in relation to suspended sediment concentration (SSC) over the study period (285 days; 2017–2024) (A). Seasonal variations in wind speed and SSC (B)

A moderate positive correlation (r = 0.55) was found between wind speed and SSC, highlighting its important role in resuspending deposited sediments into the water column (Fig. 14A). This relationship explains the elevated SSC, particularly during low stages in winter, when the highest wind speed (u_winter_ = 3.84 ± 2.19 m/s) and the lowest water depths occur (resulting in the highest bed shear stress). Additionally, during this season, the prevailing wind direction is mostly northeast and southwest, which are nearly aligned with the lake’s longest axis. These winds are more effective at resuspending deposited sediment, as they cause higher waves due to the longer fetches. This finding is consistent with the conclusions of Li et al. (2017) in their study of Lake Taihu, China. In the meantime, lower wind speeds were observed in other seasons, particularly in autumn (u_*autumn*_ = 2.48 ± 1.14 m/s).

## Spatial variations of suspended sediment concentration and vegetation coverage in Lake Tisza

The spatial distribution of SSC in Lake Tisza`s sub-basins was primarily influenced by the interplay of hydrological, geomorphological, and hydrodynamic conditions. Specifically, the highest SSC was observed in the uppermost TVM sub-basins, while the lowest occurred in the downstream AM sub-basin, with a decreasing trend along the lake (Fig. 6). This is likely attributed to the shallow and more turbulent nature of the TVM sub-basin, as well as its proximity to upstream sediment input from the river. On the contrary, the AM sub-basin is the deepest, experiencing less turbulence and receiving lower sediment input from the river. The slight decline in SSC in the PM sub-basin is likely due to its greater depth relative to the TVM and SM sub-basins (Fig. 6C). Low SSCs were observed in the TFM sub-basin due to its characteristics as a paleo-channel with deep and calm water. In the meantime, relatively high SSC was noticed in the river (i.e., TT sub-basin) due to the elevated suspended sediment load transported during flood events. Similar spatial patterns of SSC have been observed in the Xin’anjiang Reservoir, China, where SSC was highest near river inflows and declined toward deeper, calmer downstream basins (Shi et al., 2020). This trend is comparable to Lake Tisza, where the shallow, turbulent TVM sub-basin showed the highest SSC, while the deeper AM sub-basin showed the lowest, highlighting the strong role of hydrodynamics and basin morphology in shaping SSC distribution in regulated lakes.

Remarkably, this spatial pattern remained consistent across seasons, except in winter when SSC in the SM sub-basin exceeded that in the TVM sub-basin. This winter shift is probably attributed to the extremely low water levels in the lake, which drastically reduce the water volume in the TVM sub-basin. Therefore, water becomes concentrated primarily in the relatively deep paleo-channels within the sub-basin, which are characterized by lower SSC (Fig. 5D).

The correlation analysis revealed a stronger association and dependence of SSC in shallow sub-basis (i.e., SM, PM, and TVM) compared to deeper ones (i.e., AM, TFM, and TT). This finding aligns with the typical relationship between SSC and water stage in shallow tidal basins, as reported by Carniello et al. (2014). Specifically, shallow sub-basins are more sensitive to changes in water volume, which directly influence sediment resuspension, residence time, and floodplain connectivity to a greater extent than deeper sub-basins. A smaller absolute decline in water level has a greater relative impact and is more likely to cause significant sediment resuspension in shallow sub-basins than in deeper ones, due to its stronger effect on bed shear stress.

When it comes to the spatial distribution of the vegetation coverage, the greatest values were observed in the TT sub-basin, primarily due to the presence of floodplain forest along the river section. Focusing on the lake, very dense vegetation occurred in the upper TFM and TVM sub-basins, declining toward the lower AM sub-basin, which resamples the SSC distribution pattern. This trend aligns with the findings of Szabó et al. (2020), who reported high vegetation coverage, mainly consisting of water caltrop and duckweed, in shallow sub-basins (e.g., TVM), with a decline in deeper areas (e.g., AM sub-basin). Thus, the authors reported that vegetation coverage could serve as an effective indicator of water depth.

Remarkably, this spatial vegetation pattern was typical across seasons in all three studied areas (i.e., “water + non-water”, “water”, and “non-water”), aligning with findings in the upper and middle reaches of the Yellow River Basin (Gao et al., 2023). This suggests comparable environmental conditions, vegetation types, and land use across the various sub-basins, thus they respond similarly to seasonal variations.

## Sediment trapping efficiency of Lake Tisza and the river

The impoundment effect of the Kisköre Dam significantly reduces flow intensity within Lake Tisza, encouraging sedimentation and consequently diminishing storage capacity. An attempt was made to estimate the sediment trapping efficiency (STE) of the reservoir, using a simple sediment budget (Eq. 4), comparing sediment loads (*Q*_*s*_) from the uppermost section at Tiszabábolna to those just upstream of the dam (Fig. 1). Sediment discharge data for the inlet included water discharge, measured by a horizontal acoustic Doppler current profiler (H-ADCP) and SSC rating curves. In contrast, the outlet was monitored solely for water discharge. As a result, the outflow SSC was estimated using a limited set of Sentinel-2 images. However, this approach revealed substantial uncertainty.

1 $$STE \left(\%\right) =\frac{{Q}_{s,in}-{Q}_{s,out}}{{Q}_{s,in}}\times 100$$

where ${Q}_{s,in}$ and ${Q}_{s,out}$ represent the sediment discharge at the uppermost section (Tiszabábolna) and just upstream of the dam, respectively.

This uncertainty stems from both the inherent limitations in sediment measurements and remote sensing techniques, as well as the complex nature of the system. Unlike typical reservoirs, where damming impounds the river and floodplain, reducing sediment transport capacity and causing widespread sediment deposition (Julien, 2010), Lake Tisza maintains a distributed system. This unique characteristic allows the river to retain its main channel with moderate flow velocities, while connections with sub-reservoirs remain controllable.

According to Fleit et al. (2024), the flow rates in these flushing channels are more than an order of magnitude lower than in the main river. Consequently, a simple balance equation (Eq. 4) mostly captures the sediment trapping efficiency of the main channel rather than the sub-reservoirs. Additionally, the backwater effect upstream of the dam slows river flow, promoting sediment deposition within the main channel and leading to an overestimation of the sediment trapping efficiency of the sub-reservoirs.

**Fig. 15:**
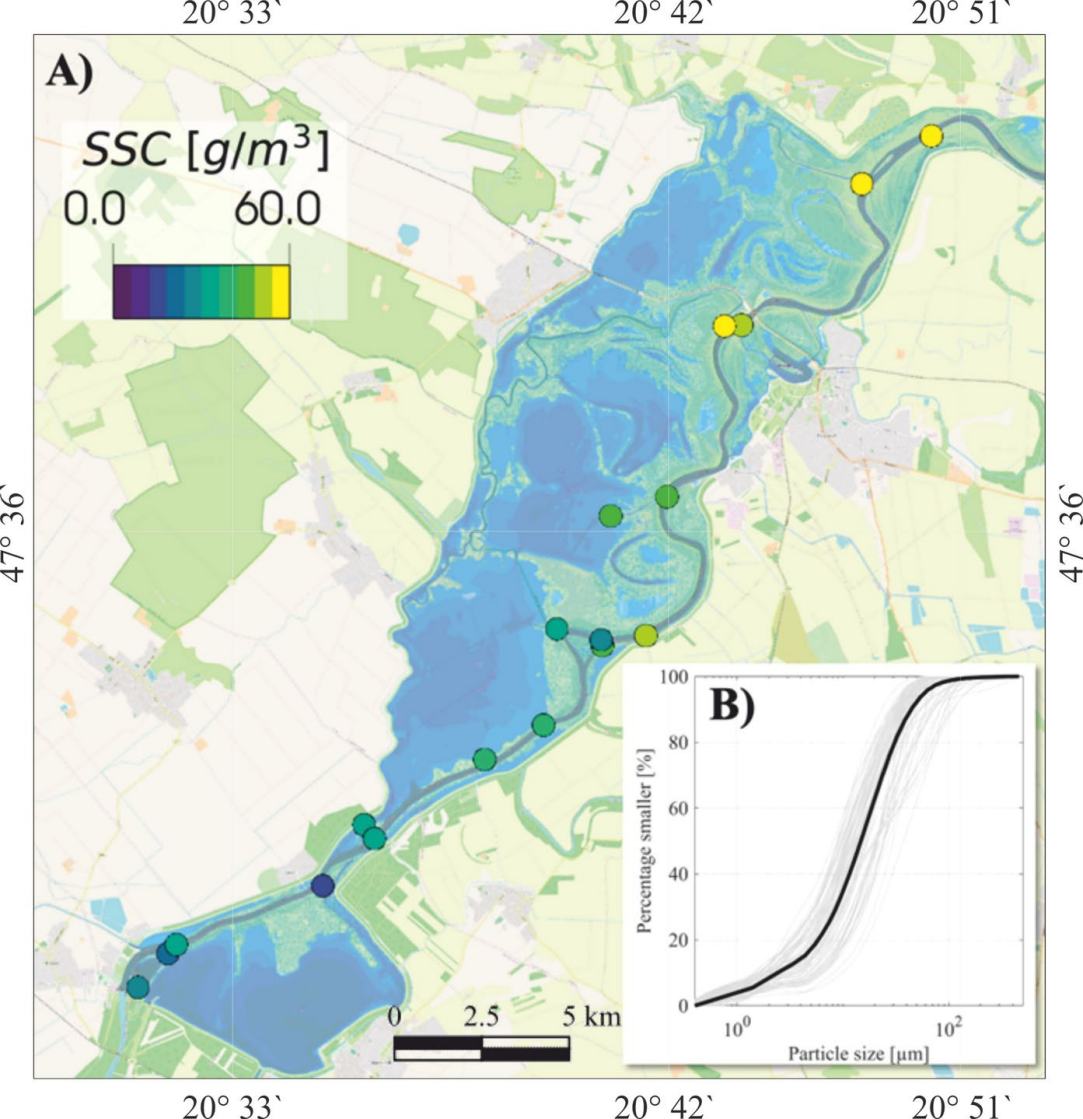
Water samples marked as disks colored based on SSC during the spring filling period and a minor flood (March, 2023) (A), grain size distribution of all suspended sediment samples. Basemap is based on © OpenStreetMap contributors (https://www.openstreetmap.org). The colormap of suspended sediment concentration (SSC) refers to the disk markers

Under regular operational conditions, mean river flow velocities are typically below 0.5 m/s throughout the studied section, dropping to below 0.3 m/s during low water conditions, with water depths reaching up to 20 m. Laboratory analyses of 68 water samples determined that the median diameter of suspended sediment particles is 15.6 ± 5.9 μm (Fig. 15B). These flow conditions favor the settling of suspended materials along the channel, as illustrated in Fig. 15.

During flood events, fine materials deposited in the main channel (Fig. 15A) become resuspended and are transported toward the dam, where they are subsequently released downstream. As a result, a substantial portion of these materials eventually exits the system. Additionally, sediment loads reaching the dam are significantly influenced by sediment discharge from the reservoir. This is particularly evident during the autumn emptying process or extended periods of severe wind-induced wave activity, which resuspend bed materials and transport them towards the dam. Such dynamics can also result in inaccurate assessments of the trapping efficiency.

These dynamic sediment movements underscore the limitations of relying solely on simple sediment budget differences for STE calculations. Achieving more reliable results necessitates a comprehensive sediment budget that accounts for all sediment inputs, outputs, and storage under varying hydrological conditions, as advocated by Dean (2005), who emphasized the importance of including uncertainty tracking and sensitivity analysis for accurate assessments of sediment fluxes and trapping efficiency. This can be effectively realized through the integration of extensive in-situ data, remote sensing techniques, and hydrodynamic modeling. Such a multifaceted approach will yield a more robust and precise understanding of sediment trapping efficiency within the reservoir system.

## Evaluation of satellite-based estimates of suspended sediment concentration and vegetation coverage in shallow lakes

The validation of the Sentinel-2-ANN-based SSC model, developed by Mohsen et al. (2023c), provided good agreement with independent in-situ SSC measurements at the validation site (Fig. 1) (R^2^ = 0.87, MAE = 21.17 g/m^3^ and RMSE = 22.67 g/m^3^), though a consistent bias was noticed. This bias might arise from different measurement techniques, or the relatively limited in-situ data used for model training, especially with the complex ANN algorithm (Ball et al., 2018). To address this, a bias correction was applied, which reduced systematic errors and provided more realistic and reliable SSC estimates. Similarly, a robust Sentinel-2-ANN-based land cover model was developed in this study for investigating riparian vegetation, with accuracy, precision, recall, and F1-score values all exceeding 0.94. The accuracy of the model was higher than its counterpart in the Lower Tisza floodplain (accuracy: 0.83), based on the decision tree (Fehérváry & Kiss, 2020), and in the Nile River islands (accuracy: 0.84), based on random forest (Kamal et al., 2022).

The performance of machine learning classification typically outweighs traditional spectral indices (e.g., NDWI and NDVI) due to their ability to handle complex spectral signatures and mixed pixels more effectively (Mohsen et al., 2023b). However, including spectral indices in model training, as an independent variable, can often enhance accuracy by improving class discrimination, particularly when classes share similar spectral signatures in the raw bands. This was evident in achieving high accuracy in detecting water pixels (Figure A4), given their clear distinction in NDWI and NDVI, while the model slightly mixes between land and vegetation. Also, water pixels have a very distinct spectral signature, while they are quite variable in vegetation and land, depending on vegetation type, health, and density, as well as land cover type (e.g., bare soil and urban area). For instance, bare soil and dry vegetation can have very similar spectral signatures, making effective discrimination between the two classes challenging (Rukhovich et al., 2023).

Overall, integrating high spatial resolution, multi-spectral Sentinel-2 images with a machine learning algorithm proved to be a highly useful and reliable approach for mapping the spatiotemporal dynamics of SSC and vegetation coverage in shallow lakes. This method can effectively provide valuable data for calibrating numerical models, which, in turn, offer practical solutions for sedimentation and eutrophication issues in such environments. However, future studies should integrate very high spatial resolution imagery (e.g., WorldView 3 and PlanetScope) and radar data to enable more in-depth analysis of aquatic vegetation and its relationship with SSC, while also addressing the limitations posed by cloud cover, particularly during flood periods. In addition, in-situ observations of aquatic vegetation are essential for calibrating and validating satellite-based models, thereby improving the discrimination of different vegetation types. Such efforts will enhance our understanding of the role of aquatic vegetation in shaping lake hydrodynamics and sediment dynamics.

## Conclusions

Machine learning algorithms, specifically ANNs, were integrated with Sentinel-2 imagery to effectively investigate the dynamics of suspended sediment concentration (SSC) and vegetation coverage in Lake Tisza in space and time. The analysis was based on a comprehensive dataset of 285 Sentinel-2 images covering the past eight years (i.e., 2017–2024). It examined the interconnected influence of hydrological, morphological, and meteorological conditions as well as the operational regime of the Kisköre Dam on suspended sediment concentration and vegetation extent.

A relatively high negative correlation occurred between SSC and regulated water levels, especially in shallow sub-basins, with the lowest concentrations observed during high water levels in summer and the highest during low levels in winter. In contrast, vegetation followed an opposite pattern. Spatial analysis demonstrated a consistent distribution of both SSC and vegetation extent across sub-basins, following a downstream decreasing trend. This highlights the uppermost sub-basins, especially the TVM sub-basin, as the most vulnerable to sedimentation and eutrophication. Moreover, the typical spatial distribution patterns of vegetation and SSC remained consistent across seasons, suggesting similar environmental conditions, vegetation types, and land use across sub-basins, leading to a uniform response to seasonal variations. Reliable estimates of sediment trapping efficiency (STE) require a comprehensive budget that accounts for the complexities of the system and the dynamic nature of sediment transport.

Remote sensing proved its potential in revealing the dynamism of SSC and vegetation extent in Lake Tisza. The integration of in-situ measurements, spectral indices, and machine learning algorithms offered robust SSC and land cover models, providing frequent, cost-effective, and reliable monitoring solutions, even for shallow lakes with complex landscape mosaics like Lake Tisza.

However, the limitations of the system should be acknowledged, including data gaps caused by cloud cover, mixed pixels, and spatial resolution constraints that reduce the accuracy of land cover classification and SSC estimates, challenges in calibration and validation due to scarce in-situ data, as well as uncertainties in estimating STE using a simplified budget model with limited inputs. Therefore, future studies integrating very high-resolution optical imagery, radar data, intensive in-situ measurements, detailed field observations of aquatic vegetation, and advanced numerical modeling are recommended to gain deeper insights into vegetation–SSC dynamics within the system.

## Figures and Tables

**Fig. 16:**
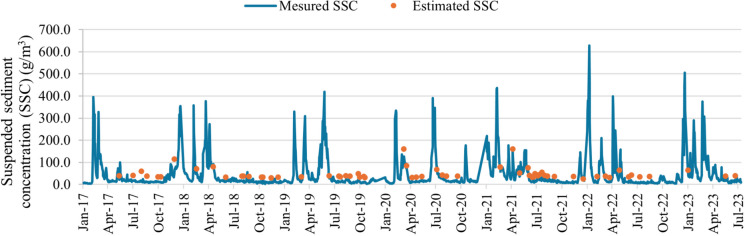
Timeseries of daily in-situ suspended sediment concentration (SSC) measurements at the validation site versus Sentinel-2 estimates on specific dates (63 concurrent dates; 2017–2023)

**Fig. 17:**
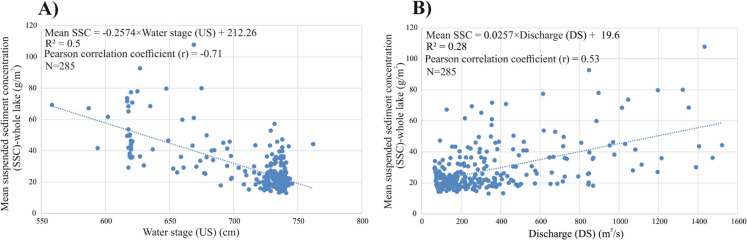
Scatter plots between the mean suspended sediment concentration (SSC) in Lake Tisza and water stage upstream (**A**) and the discharge downstream (**B**) of the dam

**Fig. 18:**
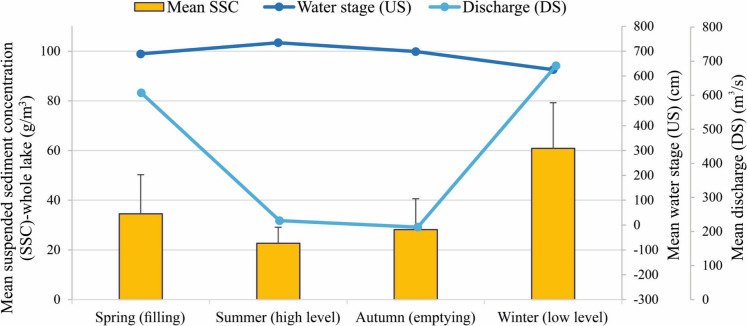
Seasonal variability of mean suspended sediment concentration (SSC) in Lake Tisza in relation to the mean water stage upstream and discharge downstream of the dam

**Fig. 19:**
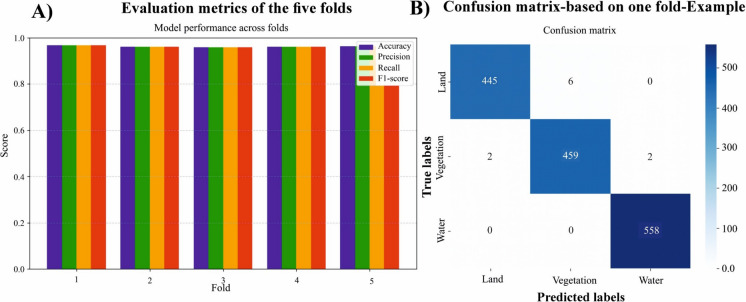
Evaluation metrics of the land cover model for the five folds (**A**). Confusion matrix based on one-fold (80% training and 20% validation) as an example (**B**)

**Fig. 20:**
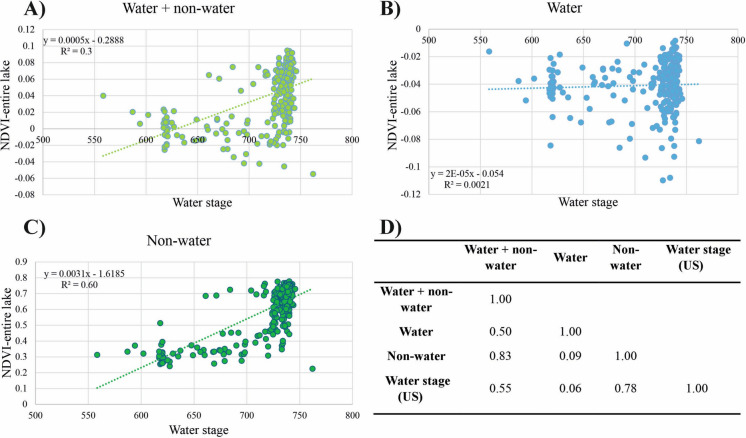
Correlation between the mean normalized difference vegetation index (NDVI) and water stage upstream, considering three areas of the reservoir—"water + non-water” (A), “water” (B), and “non-water” (C) areas. The Pearson correlation matrix among these variables is represented in panel “D”. The data are based on the analysis of 285 Sentinel-2 images from 2017 to 2024

**Fig. 21:**
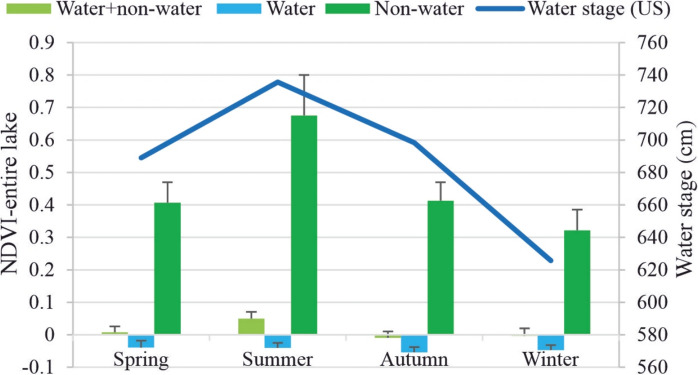
Seasonal variability of the mean normalized difference vegetation index (NDVI) for the three areas of Lake Tisza—"water + non-water”, “water”, and “non-water”—in relation to the water stage upstream. The data are based on the analysis of 285 Sentinel-2 images from 2017 to 2024

**Fig. 22:**
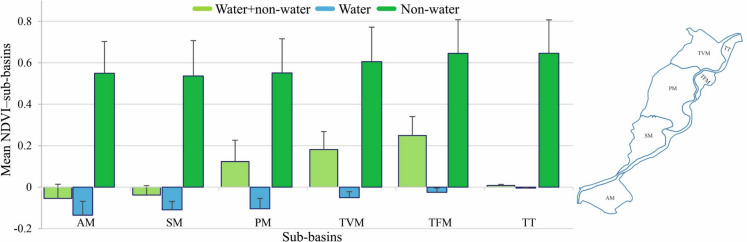
Mean normalized difference vegetation index (NDVI) of the six sub-basins, considering “water + non-water”, “water”, and “non-water” areas. The data are based on the analysis of 285 Sentinel-2 images between 2017 and 2024

**Fig. 23:**
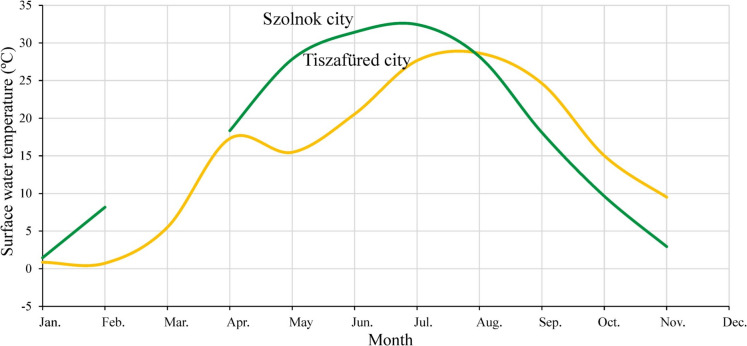
Monthly surface water temperature at channel sections near Tiszafüred (upstream of the Kisköre Dam) and Szolnok (downstream of the dam) in 2023, based on Landsat 9 estimates. Missing values correspond to months with cloud cover

**Fig. 24:**
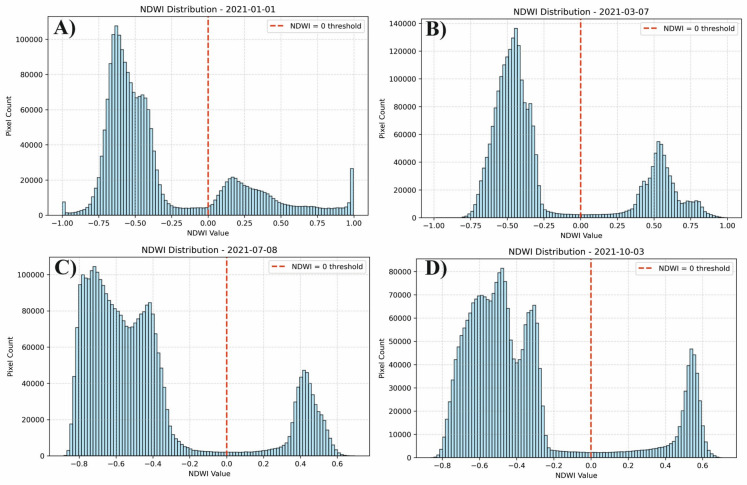
Bimodal distribution of the NDWI over Lake Tisza during different seasons of 2021, including winter (**A**), spring (**B**), summer (**C**), and Autumn (**D**)

## Data Availability

The employed data in this study are available at https://www.danubehis.org/ (accessed on January 20, 2025) and https://dataspace.copernicus.eu (accessed on January 25, 2025). All scripts developed in this study have been made publicly available at: https://github.com/AMohsenMetwaly/Lake-Tisza-SSC-Vegetation- (accessed on September 7, 2025).

## Appendix (A)

A1 $$NDWI =\frac{Green-NIR}{Green+NIR}$$

where NDWI is the normalized difference water index; Green is the green band (B3) in Sentinel-2; NIR is the near-infrared band (B8) in Sentinel-2.

A2 $$NDVI =\frac{NIR-Red}{NIR+Red}$$

where NDVI is the normalized difference vegetation index; Red is the red band (B4) in Sentinel-2.

A3 $${X}{\prime} =\frac{X-{X}_{min}}{X+{X}_{max}}$$

where $X{\prime}$ is the normalized pixel value; $X$ is the original pixel value; ${X}_{min}$ is the minimum value of $X$ in the dataset; ${X}_{max}$ is the maximum value of $X$ in the dataset. Also, the land cover classes were encoded as numerical values: land: (0), vegetation: (1), and water: (2).
